# Quinoline 3-sulfonamides inhibit lactate dehydrogenase A and reverse aerobic glycolysis in cancer cells

**DOI:** 10.1186/2049-3002-1-19

**Published:** 2013-09-06

**Authors:** Julia Billiard, Jennifer B Dennison, Jacques Briand, Roland S Annan, Deping Chai, Mariela Colón, Christopher S Dodson, Seth A Gilbert, Joel Greshock, Junping Jing, Hong Lu, Jeanelle E McSurdy-Freed, Lisa A Orband-Miller, Gordon B Mills, Chad J Quinn, Jessica L Schneck, Gilbert F Scott, Anthony N Shaw, Gregory M Waitt, Richard F Wooster, Kevin J Duffy

**Affiliations:** 1 Cancer Metabolism DPU, GlaxoSmithKline, Collegeville PA, USA; 2 Platform Technology Sciences, GlaxoSmithKline, Collegeville PA, USA; 3 Platform Technology Sciences, GlaxoSmithKline, Research Triangle Park, Chapel Hill NC, USA; 4 Department of Systems Biology, The University of Texas MD Anderson Cancer Center, Houston TX, USA

**Keywords:** LDH, Metabolism, Aerobic glycolysis, Cancer

## Abstract

**Background:**

Most normal cells in the presence of oxygen utilize glucose for mitochondrial oxidative phosphorylation. In contrast, many cancer cells rapidly convert glucose to lactate in the cytosol, a process termed aerobic glycolysis. This glycolytic phenotype is enabled by lactate dehydrogenase (LDH), which catalyzes the inter-conversion of pyruvate and lactate. The purpose of this study was to identify and characterize potent and selective inhibitors of LDHA.

**Methods:**

High throughput screening and lead optimization were used to generate inhibitors of LDHA enzymatic activity. Effects of these inhibitors on metabolism were evaluated using cell-based lactate production, oxygen consumption, and ^13^C NMR spectroscopy assays. Changes in comprehensive metabolic profile, cell proliferation, and apoptosis were assessed upon compound treatment.

**Results:**

3-((3-carbamoyl-7-(3,5-dimethylisoxazol-4-yl)-6-methoxyquinolin-4-yl) amino) benzoic acid was identified as an NADH-competitive LDHA inhibitor. Lead optimization yielded molecules with LDHA inhibitory potencies as low as 2 nM and 10 to 80-fold selectivity over LDHB. Molecules in this family rapidly and profoundly inhibited lactate production rates in multiple cancer cell lines including hepatocellular and breast carcinomas. Consistent with selective inhibition of LDHA, the most sensitive breast cancer cell lines to lactate inhibition in hypoxic conditions were cells with low expression of LDHB. Our inhibitors increased rates of oxygen consumption in hepatocellular carcinoma cells at doses up to 3 microM, while higher concentrations directly inhibited mitochondrial function. Analysis of more than 500 metabolites upon LDHA inhibition in Snu398 cells revealed that intracellular concentrations of glycolysis and citric acid cycle intermediates were increased, consistent with enhanced Krebs cycle activity and blockage of cytosolic glycolysis. Treatment with these compounds also potentiated PKM2 activity and promoted apoptosis in Snu398 cells.

**Conclusions:**

Rapid chemical inhibition of LDHA by these quinoline 3-sulfonamids led to profound metabolic alterations and impaired cell survival in carcinoma cells making it a compelling strategy for treating solid tumors that rely on aerobic glycolysis for survival.

## Background

In the presence of oxygen, most normal differentiated cells rely primarily on mitochondrial oxidative phosphorylation to generate ATP. During oxidative phosphorylation, glucose-derived pyruvate is transferred to the mitochondria and used as a substrate in the Krebs cycle. In contrast, many cancer cells have adapted to derive a significant amount of their ATP from converting glucose to lactate in the cytosol, even in the presence of oxygen, a process termed aerobic glycolysis or the Warburg effect [1]. During aerobic glycolysis, mitochondrial pyruvate uptake is suppressed and cytosolic lactate dehydrogenase (LDH) enzymes reduce this growing pyruvate pool to lactate, thereby regenerating nicotinamide adenine dinucleotide (NAD^+^) and allowing energy production to continue. LDH is a tetrameric enzyme comprised of two major subunits A and/or B, resulting in five isozymes that can catalyze the inter-conversion of pyruvate and lactate. LDHA and LDHB proteins are differentially regulated in cancer cells. LDHB, when present, is constitutively expressed, whereas LDHA is inducible in hypoxic conditions and often overexpressed in cancers with a *MYC* amplification [2]. High LDHA levels have been linked to poor prognosis in many cancer lineages [3-6]. Reduction of LDHA levels in cancer cells by siRNA or shRNA stimulates mitochondrial respiration and reduces cellular proliferative and tumorigenic potential both *in vitro* and in xenograft models [7-13].

Several small-molecule LDHA inhibitors have been described, but the potency and selectivity of these compounds are modest. Polyphenolic naphthalene FX-11 was originally reported as a potent and selective inhibitor of LDHA [14], but this activity was later corrected [8] and in our hands was modest at best (LDHA IC_50_ = 50 to 100. Several other selective LDHA inhibitors have been reported, but all have potency in the micromolar range [15-18]. Low enzymatic potency of these inhibitors makes it challenging to correlate the observed cellular effects to LDHA inhibition [8,15-17,19].

We performed a high-throughput screen followed by lead optimization to generate potent (IC_50_ = 2 to 10 nM), selective, and cell-permeable inhibitors of LDHA enzymatic activity. These compounds enabled us to explore the consequences of rapid chemical inhibition of LDHA activity in cancer cells. We found that inhibition of LDHA in hepatocellular carcinoma cells led to a rapid reduction of glucose uptake and lactate production. Comprehensive metabolic analysis revealed that the cytosolic glycolysis pathway was significantly impeded, with some intermediates increasing as much as 40-fold. As the cell lost cytosolic glucose processing capacity, Krebs cycle activity increased consistent with the hypothesis that increased cellular pyruvate is processed by the mitochondrion. Indeed, we also observed increased oxygen consumption upon LDHA inhibition. Finally, our inhibitors impaired cell survival and induced apoptosis in hepatocellular carcinoma cells.

## Methods

### Cell lines

A2780 were obtained from the European Collection of Cell Cultures (ECACC, Porton Down, UK), IGROV-1 and U251 were from the National Cancer Institute (NCI, Bethesda, MD, USA), and all other cell lines were from American Type Culture Collection (ATCC, Manassas, VA, USA). The cell lines were authenticated as previously described [20]. All cell lines were maintained in a 37**°**C 5% CO_2_-95% humidified air incubator. Breast cancer cell lines were cultured in DMEM (Life Technologies, Carlsbad, CA, USA) supplemented with 5% FBS (Sigma-Aldrich, St. Louis, MO, USA). All other cell lines were maintained in R10 medium (Roswell Park Memorial Institute medium (RPMI)-1640 (Life Technologies) supplemented with 10% FBS). MCF10A cells were supplemented with additional cholera toxin (100 ng/mL), hydrocortisone (0.5 mg/mL), insulin (10 μg/mL), and epidermal growth factor (EGF) (20 ng/mL). Stable isogenic cell lines of HCC1937 were generated using LDHA, LDHB, or the non-silencing control Expression Arrest GIPZ lentiviral shRNA particles from Open Biosystems (ThermoFisher Scientific Inc, Fremont, CA, USA). Infected cells were selected and routinely cultured with 1 μg/mL puromycin (Sigma-Aldrich).

### Materials and antibodies

Anti-LDHA rabbit monoclonal antibody, anti-cleaved poly (ADP-ribose) polymerase (PARP) (D214) mouse monoclonal antibody, and anti-pyruvate kinase M2 (PKM2) and anti-PARP rabbit polyclonal antibodies were obtained from Cell Signaling Technology (Danvers, MA, USA); anti-LDHB mouse monoclonal antibody was from Abcam (Cambridge, MA, USA); and anti-β-actin mouse monoclonal antibody was from Sigma-Aldrich. All secondary antibodies were from LI-Cor, Inc. (Lincoln, NE, USA); (1,6-^13^C_2_)glucose, oligomycin, and rotenone were from Sigma-Aldrich. All other materials were obtained from Life Technologies or from Sigma-Aldrich.

### NAD^+^/NADH concentration determination

Four million HepG2 cells were extracted with 400 μL of NAD^+^/NADH extraction buffer (BioVision Research Products, Mountain View, CA, USA) and processed with NAD^+^/NADH quantification kit (BioVision Research Products), per manufacturer’s instructions.

### Lactate production assays

Snu398 or HepG2 cells were plated at 7,500,000/mL in 100 μL R10 per well into 96-well plates and allowed to attach overnight. Compound dose responses were prepared in DMSO for nine points in 3-fold dilutions from 30 μM and added to the cells in 100 μL R10 medium. Cells were incubated for 1 h at 37**°**C to allow for inhibition of LDHA prior to start of the experiment (pre-incubation). After 1 h, the pre-incubation medium was removed, and the cells were washed in PBS and placed in 100 μL glucose-free RPMI medium supplemented with 1.7 mM glucose (low glucose medium) and containing the same compound dilutions. Cells were incubated for 30 minutes at 37**°**C and 20 μL of conditioned medium from each well was subjected to RapidFire-MS analysis using RapidFire 300 microfluidics system (Agilent Technologies, Santa Clara, CA, USA) and the API 4000 QTrap tandem mass spectrometer (Applied Biosystems, Framingham, MA, USA). Lactate was monitored by the transition of m/z 89 >m/z 43. Data were acquired using Analyst software for Windows Ver. 1.5 (Applied Biosystems), and the RapidFire Integrator 3.4 peak detection and integration software (Agilent Technologies) was used to generate peak areas for the reaction monitoring signals. The concentrations of lactate were calculated from peak areas using a lactate calibration curve. CellTiter-Fluor™ (CTF) cell viability assay (Promega, Madison, WI, USA) was used per manufacturer’s instructions to evaluate cell viability in each well, lactate concentration in each well was normalized to the CTF value, and the lactate/CTF ratio obtained in DMSO-treated cells was set at 100%. For cell lines other than Snu398 and HepG2, the cell numbers varied to obtain approximately 80% confluency and incubation times with the inhibitor varied between 30 and 120 minutes. MDA-MB-453 cells were cultured in normoxic (21% oxygen) or hypoxic (1%) conditions overnight in DMEM supplemented with 5% FBS. Medium was exchanged with physiological DMEM (5 mM glucose, 0.5 mM glutamine, no FBS) containing DMSO or Compound 1 at multiple concentrations and collected after 2 h for hypoxic cells and 6 h for normoxic cells. Lactate concentrations were quantified using a YSI 2900 Biochemistry Analyzer with a lactate oxidase probe (YSI Incorporated, Yellow Springs, OH, USA).

### ^13^C nuclear magnetic resonance (NMR) spectroscopy

Snu398 or HepG2 cells were trypsinized and 2,000,000 cells were resuspended in 10 mL R10 containing LDHA inhibitor or DMSO control and incubated for 1 h at 37**°**C to ensure LDHA inhibition prior to start of NMR data acquisition. After this pre-incubation, cells were washed in PBS and resuspended in 400 μL of glucose-free RPMI supplemented with 10% deuterium oxide, 11 mM D-(1,6-^13^C_2_)glucose, and LDHA inhibitor or DMSO. An additional 600 μL of the same medium was gently added to top off the resuspended cells. Five mm NMR tubes with reduced sample depth (Shigemi Inc, Allison Park, PA, USA) were used to ensure that the cells remain inside the active volume of the receiver coil of the probe. The ^13^C NMR spectra were acquired with a Varian Unity INOVA™ 600 system (Agilent Technologies) operating at 150.8 MHz. The spectra were recorded using a 5-mm broadband probe with ^1^H decoupling, regulated at 37**°**C, 32,000 complex data points, sweep width of 37559 Hz, acquisition time of 0.8724 sec/scan, 128 scans and a relaxation delay of 1 sec. Prior to Fourier transformation, sensitivity and resolution were enhanced by applying an exponential line-broadening of 1 Hz over the first 32,000 data points and then zero filled to 256,000 data points. Spectra were analyzed using in-house data analysis software.

### Oxygen consumption and extracellular acidification rates measurement

One hundred twenty thousand Snu398 or HepG2 human hepatocellular carcinoma cells per well were plated in XF24 PS or XF96 PET cell culture microplates (Seahorse Biosciences, North Billerica, MA, USA) and allowed to attach and proliferate for 18 h. Cells were equilibrated with DMEM lacking bicarbonate (Seahorse Biosciences) supplemented with glucose at 37**°**C for 1 h in an incubator lacking CO_2_. The rate of change of dissolved O_2_ (oxygen consumption rate, OCR) and rate of change of pH (extracellular acidification rate, ECAR) in medium immediately surrounding the cells was measured in the XF24 Analyzer (Seahorse Biosciences) per manufacturer’s instructions. Compound doses were injected into the wells at the indicated times. For dose response curves, the OCR and ECAR levels obtained in each well before compound addition were subtracted from the values obtained 92 minutes after compound addition.

For XF96 experiments breast cancer cells were seeded in DMEM without bicarbonate at 16,000/well in XF96 V3 PET plates (Seahorse Biosciences) coated with CellTak (BD Biosciences, San Jose, CA, USA). After baseline readings were obtained, multiple concentrations of compound 1 (2.5 to 40 μM) or DMSO, rotenone (1 μM), and antimycin (1 μM) were added sequentially. HepG2 cells were seeded at 16K/well in XF96 V3 PET plates and left overnight for attachment. The medium was exchanged with DMEM without bicarbonate prior to readings on the XF96 Analyzer (Seahorse Biosciences).

### Seahorse XF96 permeabilized cells

HepG2 cells were seeded overnight in DMEM, 10% FBS, at 16,000 cells/well in an XF96 V3 PET plate. The next day, the medium was removed, and the wells were rinsed and filled with mitochondrial assay solution (MAS: 70 mM sucrose, 220 mM mannitol, 10 mM KH_2_PO_4_, 5 mM MgCl_2_, 2 mM HEPES, 1.0 mM EGTA) containing 10 mM pyruvate/10 mM malate/4 mM ADP and 2 nM XF Plasma Membrane Permeabilizer (PMP) (Seahorse Biosciences). Compound 1, oligomycin (2 μg/mL), and antimycin (4 μM) were added sequentially. OCR and ECAR readings were determined for three cycles (two minutes mixing, two minutes measuring) after each compound addition.

### Metabolomics analysis

Ten million Snu398 or HepG2 cells per flask were plated in R10 medium in T150 tissue culture flasks and allowed to attach overnight. The following morning, medium was replaced with medium containing either DMSO or 10 μM LDHA inhibitor, and cells were incubated for 24 h at 37**°**C. The conditioned medium was collected and cells were washed, lifted by trypsinization and pelleted. Both medium and cell samples were submitted for mass spectroscopy (MS) analysis of over 500 individual metabolites. All samples were prepared in replicates of five.

### Pyruvate kinase (PK) activity and PKM2 tetramerization determination

One and a half million cells per well were plated in 6-well tissue culture plates in R10 medium and allowed to attach overnight. The following morning, cells were washed and medium replaced with 2 mL/well of low-glucose medium. DMSO control or the indicated doses of LDHA inhibitor dissolved in DMSO were added to the wells and cells were incubated at 37**°**C for 6 h. Following the incubation, cells were washed, scraped into 200 μl of distilled de-ionized water and homogenized on ice using OMNI THq homogenizer (Omni International, Kennesaw, GA, USA). Homogenates were cleared by centrifugation and protein concentration measured by bicinchoninic acid (BCA) assay (Thermo Fisher Scientific, Waltham, MA, USA). PK activity was determined using PK Activity kit (BioVision Research Products) per manufacturer’s instructions and normalized to protein concentration. For detection of PKM2 oligomerization, the same protein samples were separated by non-denaturing gel electrophoresis using the NativePAGE™ gel system (Life Technologies), transferred onto polyvinylidene difluoride (PVDF) membranes and subjected to western immunoblotting with anti-PKM2 antibody.

### Cell proliferation and survival analysis

Sixty thousand Snu398 cells per well were plated in 6-well tissue culture plates in RPMI-1640 medium supplemented with 2.5% charcoal-stripped FBS (Life Technologies). Cells were allowed to attach overnight and then DMSO control or the indicated doses of LDHA inhibitor dissolved in DMSO were added directly to the wells. After 4 to 8 days of incubation in the indicated oxygen conditions, adherent cells were trypsinized, counted, and had their viability assessed by the trypan-blue exclusion method using the Vi-Cell XR Cell Viability Analyzer (Beckman Coulter, Brea, CA, USA). The same procedure was carried out for HepG2 cells, except non-charcoal-stripped FBS was used. For PARP cleavage analysis, both floating and adherent cells were collected after 24 h and the whole-cell extracts were prepared in radioimmunoprecipitation assay (RIPA) buffer (Sigma-Aldrich) supplemented with protease and phosphatase inhibitor cocktails (Sigma-Aldrich). Solubilized proteins were separated by SDS-PAGE, transferred onto nitrocellulose membrane, and detected with each specific antibody.

### Pathway contribution analysis

Gene sets covering selected pathways were identified using published resources [21] and were as follows: glycolysis - *GPI, TPI1, PFKL, ALDOB, PKLR, PGAM1, HK1, PGK1, GAPDH, ENO1*; fatty acid synthesis - *PTGES3, PRG3, FADS1, PTGS1, MCAT, FADS2, CD74, BRCA1, MIF, OXSM, PTGDS, LTA4H, HPGD, DEGS1*; glutaminolysis - *GCLC, ALDH18A1, GLUD2, GLUD1, ASL, GCLM, ARG1, PYCR1, GAD2, ALDH4A1, ASRGL1, DDAH2, GAD1, DDAH1*; pentose phosphate - *ALDOA, TALDO1, ALDOC, PGD, ALDOB, TKTL2, TKTL1, DERA, RPIA, PRPS1L1, PFKL, PFKP, FBP1, TKT, RBKS, PFKM, FBP2, LOC729020, PGM2, GPI, PGLS, RPE, G6PD, H6PD, PGM1, PRPS2, PRPS1*. Increases in transcript levels of the enzymes composing these cellular processes were used as measurements of pathway activation. To this end, transcript levels were obtained from data that we previously submitted to the public database (https://cabig-stage.nci.nih.gov/community/caArray_GSKdata/), converted to *z*-scores, and summed over the pathway for each cell line. The total sum served as a measurement of pathway activity.

### PK analysis

All studies were conducted after review by the Institutional Animal Care and Use Committee at GlaxoSmithKline (GSK) and in accordance with the GSK Policy on the Care, Welfare and Treatment of Laboratory Animals. Compound 1 was administered to male CD mice or male Sprague–Dawley rats orally or by intravenous (IV) infusion over 120 minutes into a femoral vein. Arterial blood samples were collected over time and Compound 1 concentration was determined by liquid chromatography (LC)/MS/MS analysis.

### Statistical analysis

Statistical significance was determined using two-way analysis of variance (ANOVA) with contrasts for pairwise comparisons. Results were considered statistically different when *P* was <0.05.

## Results

### Quinoline 3-sulfonamides inhibit LDHA and reduce lactate production in cancer cells

**Figure 1:**
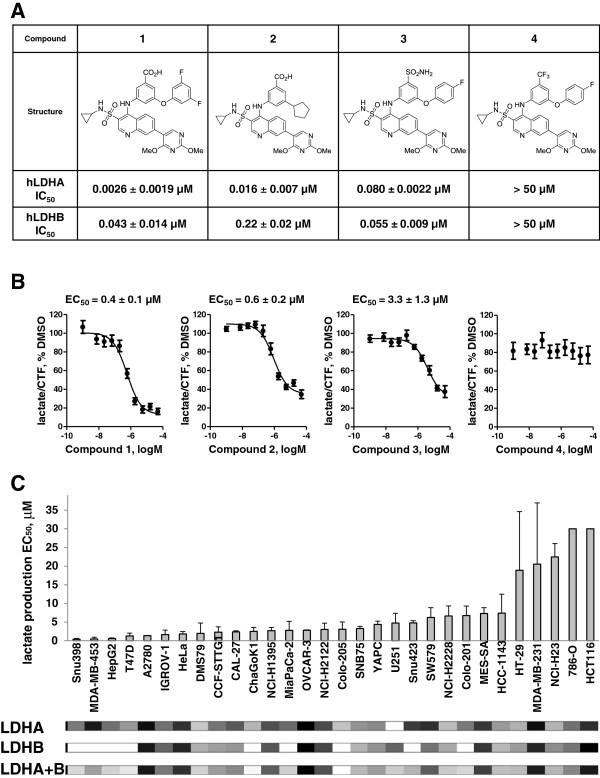
**Quinoline 3-sulfonamides inhibit lactate dehydrogenase A (LDHA) and reduce lactate production in cancer cells. (A)** Structures of the LDHA inhibitors and their potency on recombinant human LDH enzymes. **(B)** Potent LDHA inhibitors (Compounds 1 to 3) inhibit lactate production in Snu398 hepatocellular carcinoma cells whereas an analog that does not have LDHA inhibitory activity (Compound 4) does not affect cellular lactate. Lactate concentration in the conditioned medium was normalized to cell viability assessed by CellTiter Fluor™ assay (CTF), and the lactate/CTF ratio obtained in dimethyl sulfoxide -treated cells was set at 100%. Data are means ± standard error (SE) of at least five independent experiments with two replicates each. Half maximal effective concentrations (EC_50_) are means ± SE of at least five independent experiments. **(C)** Cancer cell lines exhibit different sensitivity to LDHA inhibition. Thirty-one cancer cell lines of different origins were analyzed in the lactate production assay described in **(B)**. Data are means ± SD of at least two independent experiments with 2 replicates each. Heat maps of LDHA, LDHB, and combined transcript expression obtained from gene expression analysis are shown underneath the graph, with the darker color representing higher expression. Expression values in MAS5 units are listed in Additional file 3. MAS5 units are obtained by processing the data using Affymetrix MAS5 algorithm, with target value set at 100.

To identify small-molecule inhibitors of LDHA, we developed an assay where recombinant human LDHA or LDHB enzymes catalyzed conversion of lactate to pyruvate, and the level of NADH produced in this reaction was measured through the conversion of resazurin to resorufin by diaphorase. This assay was used to screen the GSK proprietary collection of compounds and the hits identified were clustered by chemical similarity, screened to ensure no interference with the coupling reaction, and tested for stabilizing LDHA in a thermal shift stability assay [22]. From this campaign, we identified 3-((3-carbamoyl-7-(3,5-dimethylisoxazol-4-yl)-6-methoxyquinolin-4-yl)amino)benzoic acid as an NADH-competitive LDHA inhibitor. Subsequent lead optimization (Duffy *et al*., in preparation) yielded molecules with LDHA inhibitory potencies as low as 2 to 3 nM and selectivity over LDHB on the order of 10- to 80-fold (Compounds 1 to 3, Figure 1A, and Compound 5, in Additional file 1: Figure S1). Given that NAD^+^ concentration in the assay (150 μM) was equal to its K_m_, these potencies correspond to apparent inhibition constant K_i_ = IC_50_/(1 + [S]/K_m_) = 4–6 nM. These molecules did not have any appreciable activity against a panel of 32 common enzymes, receptors, and ion channels (Additional file 2: Table S1). The only appreciable activity observed was as inhibitors of phosphodiesterase 4B, but with 30- to 500-fold lower potency than for LDHA.

To test cellular activity of these inhibitors, Snu398 human hepatocellular carcinoma cells with undetectable levels of LDHB protein (Additional file 1: Figure. S4A) were treated with increasing doses of the inhibitors. Lactate concentration in the conditioned medium was assessed by RapidFire-MS analysis. Profound inhibition of cellular lactate production was observed by active LDHA inhibitors, Compounds 1 to 3, but not by an inactive analog, Compound 4 (Figure 1B). However, inhibitors with 2 to 16 nM potency against the recombinant enzyme inhibited cellular lactate production with half maximal effective concentrations (EC_50_) of 400 to 600 nM. To investigate whether low cellular permeability contributed to high cellular EC_50_, cytosolic extract was prepared from Snu398 cells treated with 1 μM Compound 2 and compound concentration in this extract was assessed by LC/MS/MS analysis. The apparent intracellular concentration of Compound 2 was 18 μM, suggesting that poor cellular penetration did not account for the observed loss of activity. Compounds 1 and 3 were also shown to have higher apparent intracellular concentrations than that of the medium (Additional file 1: Figure S2A). These compounds are likely highly protein bound in the cytosol, given that they exhibit high binding in a rat plasma protein equilibrium dialysis assay (98.8 to 99.97% for the compounds tested). Thus, while the compounds appear to enter the cells, high apparent intracellular concentrations do not necessarily indicate high unbound intracellular concentrations.

Since our inhibitors are NADH-competitive, it is possible that lower cellular potencies are driven by high cellular concentrations of NADH. We therefore determined concentrations of NAD^+^ and NADH in cells and found them to be 75 and 18 μM, respectively. While the potency of Compound 2 was dependent on NADH concentration in a cell-free biochemical assay (Additional file 1: Figure S2B), the compound IC_50_ was still only 67 nM, even at a high concentration of NADH (120 μM), indicating that intracellular levels of NADH alone do not explain the loss of compound potency. Another reason cellular EC_50_ may be higher than the enzymatic potency of the compound is if the amount of LDHA protein in the cell exceeds the K_i_ of the compound. Indeed, cellular EC_50_ = K_i_ + 1/2E_t_, where E_t_ is the cellular concentration of the enzyme. In most cases, K_i_ far exceeds the cellular E_t_ of the target and EC_50_ is approximately equal to the K_i_. However, if E_t_ is equal to or greater than the K_i_ of the inhibitor, this assumption is invalid. To test whether the E_t_ of LDHA was close to the K_i_ of our LDHA inhibitors, we used an absolute stable isotope labeling by amino acids in cell culture (SILAC) method (Additional file 1: Figure S3 [23]) to quantify LDHA in cells and found that Snu398 cells expressed 2.9 million copies of LDHA per cell, corresponding to 1.5 μM. Thus cellular EC_50_ observed for our LDHA inhibitors could be explained by high concentration of LDHA present in Snu398 cells. We next investigated LDHA expression in other cell lines and in primary human cancer tissues. Evaluation of gene expression analysis data for 428 cancer cell lines that we previously submitted to the public database (https://cabig-stage.nci.nih.gov/community/caArray_GSKdata/) identified only three lines with LDHA transcript abundance around 300 MAS 5 units, with all other lines ranging from 2,000 to 8,000. The LDHA protein expression in select lines was tested by western immunoblotting alongside Snu398 cells and agreed with gene expression data (Additional file 1: Figure S4A, C). The U251 glioblastoma line did not express detectable levels of LDHA, whereas all other lines tested expressed micromolar levels of LDHA protein. Similarly, we found high LDHA expression levels across primary human cancer tissues (Additional file 1: Figure S4B, D). LDHB transcript abundance ranged from 30 MAS 5 units (the assay detection limit) to 7,000, with the cell lines with low-to-zero LDHA protein expressing a considerable amount of LDHB (Additional file 1: Figure S4A).

We screened 30 cancer cell lines with different LDHA and LDHB expression levels in the lactate production assay (Figure 1C, see Additional file 3: Table S2 for cell line information). The potency of Compound 1 across these lines ranged from 400 nM to no effect (EC_50_ reported as 30 μM, which is the highest dose used in the assay). For the cell-line panel tested, Compound 1 potency did not correlate with LDHA, LDHB, or the total LDH expression levels (Figure 1C, Additional file 3: Table S2).

### Breast cancers with low LDHB expression are most sensitive to LDH inhibition by quinoline 3-sulfonamides in hypoxia

**Figure 2:**
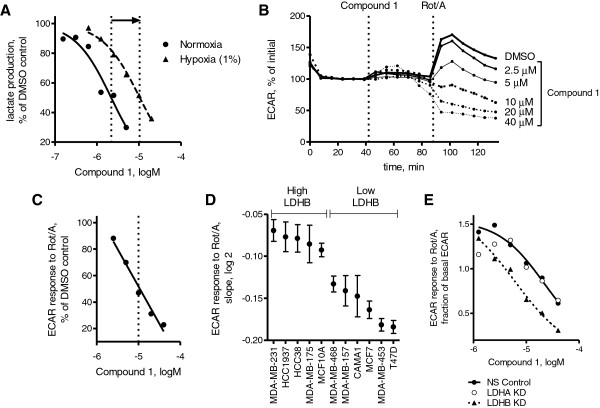
**Effects of Compound 1 on lactate production in hypoxia/anoxia and the role of lactate dehydrogenase B (LDHB) expression in breast cancer cells. (A)** Effect of hypoxia on lactate production EC_50_ values of Compound 1. MDA-MB-453 cells were cultured in normoxic (21% oxygen) or hypoxic (1%) conditions overnight. Medium was exchanged with physiological DMEM containing dimethyl sulfoxide (DMSO) or Compound 1 at multiple concentrations and collected after 2 h for hypoxic cells and 6 h for normoxic cells. EC_50_ values were estimated based on a 50% reduction in lactate production (dotted lines). **(B)** Extracellular acidification rate (ECAR) as a measure of lactate production after mitochondrial inhibition was quantified for MDA-MB-453. The baseline ECAR reading was obtained, and multiple concentrations of Compound 1 (2.5 to 40 μM) or DMSO were added followed by rotenone (Rot) (1 μM) and antimycin (1 μM). ECAR reading immediately prior to Compound 1 injection was set at 100%. **(C)** ECAR response of MDA-MB-453 cells to rotenone/antimycin after Compound 1 addition from **(B)** as a function of Compound 1 concentration. The averages of the final three readings were normalized to the untreated DMSO control. EC_50_ value was estimated based on a 50% reduction in ECAR. **(D)** Ssensitivity of a panel of breast cancer cell lines to Compound 1 after mitochondrial inhibition. Log_2_-linear slopes of ECAR response (obtained as in **(C)** for MDA-MB-453) were estimated for multiple cell lines between 2.5 and 40 μM. Means ± standard error (SE) of fitted values are shown. Cells were classified as low LDHB if the primary LDH tetramer was LDH5. Relative LDHB expression was determined previously by non-denatured electrophoresis [25]. **(E)** Effect of LDHA or LDHB expression on Compound 1 sensitivity in HCC1937 cells. Stable isogenic lines were created using a lentiviral shRNA (non-silencing. LDHA, or LDHB) with puromycin selection.

We then asked whether Compound 1 could prevent the increased lactate flux caused by metabolic compensation during hypoxia. MDA-MB-453 human breast cancer cells were chosen for these experiments because they demonstrated a 4-fold increase in lactate production in hypoxic conditions. Compound 1 inhibited lactate production in hypoxia but at higher concentrations than in normoxia (Figure 2A, EC_50_ = 10 μM). To mimic acute hypoxic/anoxic response of cells, rotenone and antimycin were used to inhibit mitochondrial function. Rapid changes in lactate production were determined by measuring ECAR with the Seahorse analyzer (Figure 2B). Compound 1 reduced ECAR with EC_50_ = 10 μM (Figure 2C) similar to that obtained for lactate flux in hypoxic conditions (Figure 2A). Since LDHB is differentially expressed in breast cancers and may be essential for proliferation [24], we investigated if LDHB expression affects sensitivity of breast cancer cells to Compound 1. We quantified the log-linear slopes of the ECAR dose–response curves in 11 breast cancer lines and determined that cancers with low LDHB expression were the most sensitive to Compound 1 (Figure 2D). To further evaluate whether LDHB expression promoted resistance to Compound 1 inhibition, isogenic cell lines with stable LDHB knockdown were created using HCC1937, a breast cancer cell line with more than 50% of its LDH activity derived from LDHB [25]. Consistent with the breast cell-lines panel results, the HCC1937 cells with LDHB knockdown were more sensitive to Compound 1 inhibition (Figure 2E). Stable knockdown of LDHA in HCC1937 cells did not change their sensitivity to Compound 1 inhibition (Figure 2E), suggesting that the observed shift in dose response upon LDHB knockdown did not result from a reduction in total LDH activity.

### Quinoline 3-sulfonamides reduce glucose uptake and increase mitochondrial oxygen consumption

**Figure 3:**
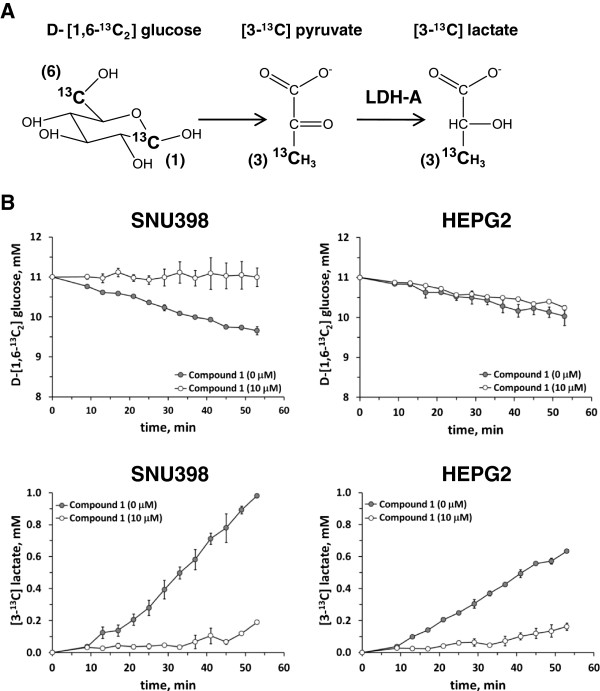
**Real-time **^**13**^**C nuclear magnetic resonance (NMR) spectroscopy analysis of D-(1,6-**^**13**^**C**_**2**_**)glucose consumption and (3-**^**13**^**C)lactate production for Snu398 and HepG2 human hepatocellular carcinoma cells. (A)** Selective ^13^C-enrichment of glucose enables detection of ^13^C-enriched metabolites originating exclusively from glucose metabolism. **(B)** Time-dependent changes in concentrations of D-(1,6-^13^C_2_)glucose and (3-^13^C)lactate obtained by recording NMR spectra every 4 minutes in Snu398 and HepG2 cells in the absence and presence of 10 μM Compound 1. Peak areas for ^13^C-1 of glucose and ^13^C-3 of lactate were used for measuring their concentrations. Data are means ± SD of two independent experiments.

Two hepatocellular carcinoma cell lines where Compound 1 had high potency - Snu398 and HepG2 - were selected for more detailed metabolic studies. Both of these lines expressed micromolar levels of LDHA (2.6 μM for HepG2 cells as determined by absolute SILAC) but no LDHB (Additional file 1: Figure S4A). Glucose utilization in Snu398 and HepG2 cells was evaluated non-invasively by real-time ^13^C NMR spectroscopy of ^13^C-labeled glucose (Figure 3A). Using an equal number of cells, Snu398 cells consumed glucose more rapidly and produced more lactate than HepG2 cells (Figure 3B). Addition of inhibitor significantly slowed down lactate production in both lines but only affected glucose consumption in Snu398 cells; (3-^13^C)pyruvate was not detected in either line.

**Figure 4:**
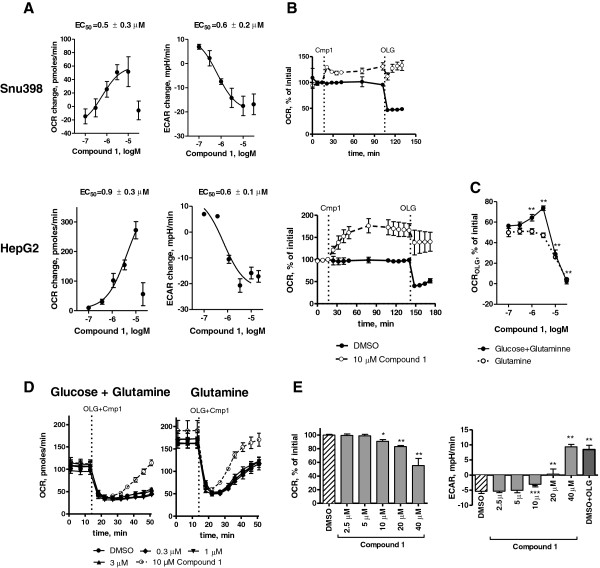
**Effects of Compound 1 on extracellular acidification rate (ECAR) and oxygen consumption rate (OCR). (A)** Change in ECARand OCR of Snu398 (top) and HepG2 (bottom) cells upon addition of lactate dehydrogenase A (LDHA) inhibitor. ECAR and OCR values were assessed by Seahorse XF Analyzer before and after addition of Compound 1, and changes observed 92 minutes after inhibitor addition were plotted as a function of inhibitor concentration. Results are means ± standard error (SE) of at least three independent experiments containing four wells per condition. EC_50_ values are means ± SE of at least three independent experiments. **(B)** Time-dependent changes in OCR in Snu398 (top) and HepG2 (bottom) cells in response to Compound 1 addition followed by 1 μg/mL oligomycin addition normalized to basal values. **(C)** Oligomycin-dependent OCR (OCR_OLG_) of HepG2 cells as a function of Compound 1 concentration. OCR_OLG_ is defined as the OCR change to oligomycin following Compound 1 addition. Cells were measured in 5 mM glucose/0.5 mM glutamine or 0.5 mM glutamine DMEM. Values are presented as a percentage of the basal OCR. **(D)** OCR of HepG2 cells in response to Compound 1 in DMEM containing 5 mM glucose and 0.5 mM glutamine (left) or 0.5 mM glutamine alone (right). Oligomycin (1 μg/mL) and Compound 1 were added simultaneously after 14 minutes, and the time-dependent OCR was recorded. Residual OCR after oligomycin addition represents the non-mitochondrial and mitochondrial proton leak contributions to the total OCR. Non-mitochondrial OCR as determined by antimycin addition was unchanged after Compound 1 addition (data not shown). **(E)** OCR and ECAR responses of HepG2 mitochondria to Compound 1. Cells were permeabilized using 2 nM PMP in MAS containing 10 mM pyruvate/10 mM malate/4 mM ADP. For **(B-E)**, each point represents means ± SD. ^*^P <0.05, ^**^P <0.01, ^***^P <0.001 compared to untreated controls.

To address possible changes in mitochondrial activity due to LDHA inhibition, we measured OCR and ECAR in Snu398 and HepG2 cells. Except for the highest dose, we observed a dose-dependent increase in OCR and decrease in ECAR in both cell lines in response to Compound 1 (Figure 4A). These effects reached steady state approximately 30 minutes after compound addition (Figure. 4B). To determine whether the increase in OCR by Compound 1 resulted in increased ATP production, oligomycin was added to inhibit ATP-synthase. At concentrations of 1 to 3 μM, we confirmed that increased OCR was used to produce additional ATP as shown by increased oligomycin-dependent OCR (OCR_OLG_) (Figure 4C). These results are consistent with the requirements of the cell to produce ATP via oxidative phosphorylation when pyruvate and NADH cannot be processed by LDH. Also consistent with this hypothesis, increased OCR_OLG_ was not observed when glutamine alone was utilized as a substrate (Figure 4C). However, OCR_OLG_ was reduced at 10 μM Compound 1 (Figure 4B), suggesting additional effects were observed on mitochondrial function. At 10 μM Compound 1, mitochondrial proton leak increased and OCR_OLG_ decreased independently of glucose flux via glycolysis, given that equivalent responses were observed using glutamine without glucose (Figure 4C-D).

To verify direct mitochondrial effects of Compound 1 at higher doses, experiments were performed with permeabilized HepG2 cells using pyruvate/malate as substrates to evaluate ADP-coupled mitochondrial function separately from cytosolic LDH function. No changes in OCR or ECAR were observed at concentrations up to 3 μM suggesting that cytosolic actions of the compound, presumably as an LDHA inhibitor, were required for OCR and ECAR changes in intact cells. However, doses of 10 μM or higher reduced mitochondrial respiration as shown by decreased OCR and increased ECAR that are likely unrelated to LDH inhibition (Figure 4E). Increases in mitochondrial ECAR reflect reduced mitochondrial proton uptake in permeabilized cells [26]. Additional analyses demonstrated that oligomycin-sensitive OCR was reduced, and proton leak increased (Additional file 1: Figure S5). Total inhibition of ATP-coupled respiration was observed at the highest dose, given that ECAR changes were equivalent to inhibition of ATP-synthase by oligomycin. Given that Compound 1 exhibits mitochondrial effects in permeabilized cells at 10 μM and that it accumulates inside cells (Additional file 1: Figure S2A), it may appear surprising that we do not observe off-target mitochondrial effects in intact cells at doses below 10 μM in the medium. However, the intracellular unbound fraction of Compound 1 available for mitochondrial inhibition may be substantially lowered by cytosolic protein binding, given that Compound 1 is highly bound to both rat and human plasma proteins (98.8 and 98.7%, respectively, by equilibrium dialysis). Therefore, permeabilized cell experiments indicate that Compound 1 is likely to have direct mitochondrial effect, but these experiments could not establish the dose at which these effects are likely to manifest in intact cells. It should be noted that direct mitochondrial effects have been observed with other drugs with well-defined mechanism of action, such as the topoisomerase inhibitor, etoposide [27].

### LDH inhibition by quinoline 3-sulfonamides alters multiple metabolic pathways in Snu398 cells

**Figure 5:**
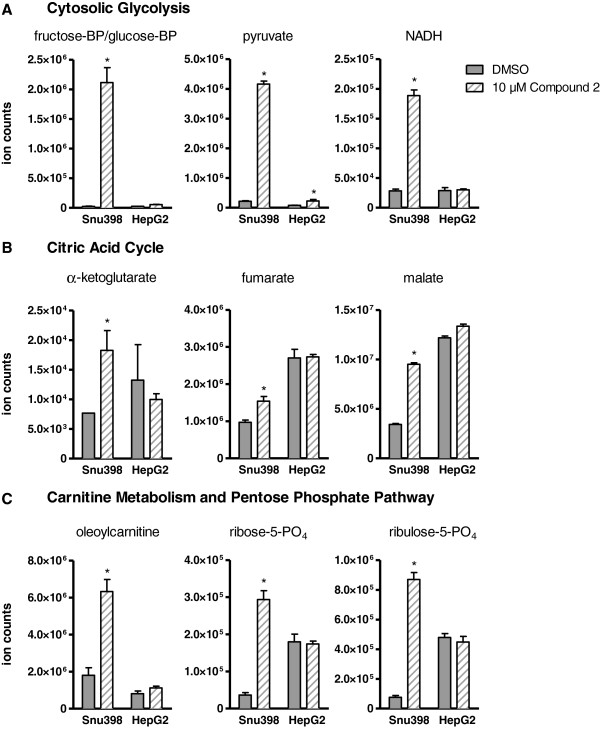
**Lactate dehydrogenase A (LDHA) inhibitor induces profound metabolic changes in Snu398, but not HepG2 hepatocellular carcinoma cells.** Cells were treated with 10 μM of Compound 2 or dimethyl sulfoxide (DMSO) control for 24 h and whole-cell extracts and conditioned media were subjected to mass spectrometry analysis of over 500 metabolites. Changes in ion counts observed in the whole-cell extracts in select intermediates of cytosolic glycolysis **(A)**, citric acid cycle **(B)**, and carnitine metabolism and pentose phosphate pathway **(C)** are shown. Data are presented as means ± standard error SE of five replicates per condition, ^*^P ≤0.001. Changes in all other metabolites in both cell lines and conditioned media are presented in Additional file 3.

We next defined the metabolic signature of LDHA inhibition. Snu398 and HepG2 cells were incubated with or without 10 μM of Compound 2 for 24 h and whole-cell lysates and extracellular media were collected and subjected to MS analysis for more than 500 metabolites (Additional file 4: Table S3 and Additional file 5: Table S4). These data demonstrate that after 24 h, continuous LDHA inhibition had caused profound metabolic changes in Snu398, but not in HepG2 cells (Figure 5). The cytosolic glycolysis pathway in Snu398 cells was impeded, with multiple intermediates increasing, some as much as 40-fold. In contrast, only modest changes in pyruvate and no other glycolytic intermediate increases were observed in HepG2 cells. As cytosolic glucose processing became inhibited, the concentrations of Krebs cycle intermediates increased, with three out of five measured metabolites increasing significantly. Taken together with the OCR measurements, this supports the hypothesis that pyruvate enters mitochondria and restores activity resulting in increased oxygen consumption upon LDHA inhibition. No change in Krebs cycle intermediates was observed in HepG2 cells, suggesting that the acute change in OCR seen in these cells upon LDHA inhibition does not translate into a long-term effect on HepG2 metabolism. Several pathways that rely on glycolytic and Krebs cycle intermediates were also upregulated in Snu398 cells, including carnitine metabolism and the pentose phosphate pathway. Once again, all these changes were restricted to Snu398 cells. There were almost no changes observed in conditioned media from either cell line (Additional file 5: Table S4, Additional file 6). Among very few statistically significant changes, we observed a 3-fold increase in extracellular pyruvate in Snu398 cells, but not in HepG2 cells. Increase in fructose-1,6-bisphosphate (FBP) in Snu398 cells was confirmed using MS analysis upon LDHA inhibition by 10 μM Compound 1 (Additional file 1: Figure S6).

**Figure 6:**
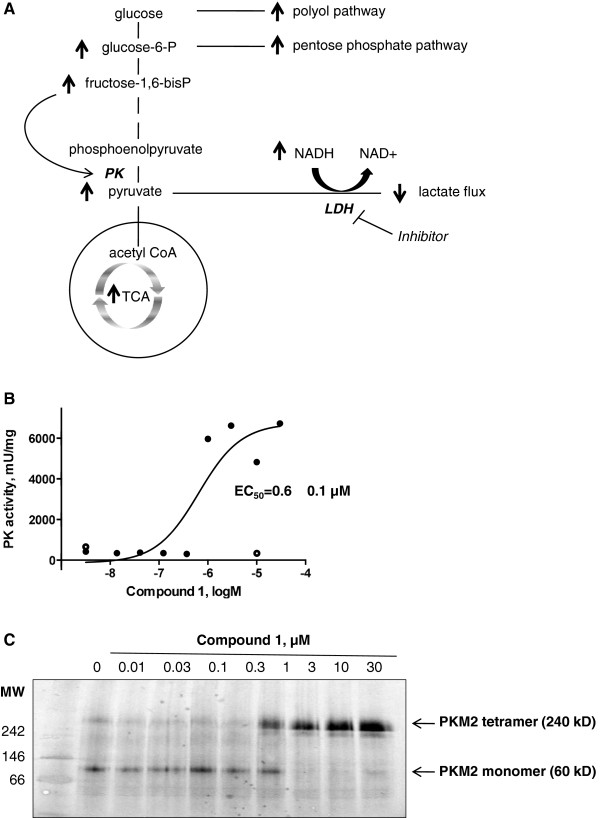
**Compound 1 causes increase in pyruvate kinase (PK) activity. (A)** Schematic representation of the metabolic changes observed in Snu398 cells upon LDHA inhibition indicating a significant rise in fructose-1,6-bisphosphate that is known to potentiate PK activity. **(B)** Compound 1 causes dose-dependent increase in PK activity in Snu398, but not HepG2 cells. Cells were incubated with dimethyl sulfoxide (DMSO) or increasing doses (Snu398 cells, closed circles) or 10 μM (HepG2 cells, open circles) of Compound 1 for 6 h and whole-cell extracts were isolated and subjected to PK activity analysis. **(C)** Compound 1 causes dose-dependent increase in Pyruvate kinase M2 (PKM2) tetramerization in Snu398 cells. Snu398 cells were incubated with DMSO control or increasing doses of Compound 1 for 6 h and whole-cell extracts were isolated and subjected to native gel electrophoresis followed by western immunoblotting with PKM2-specific antibody. NAD^+^, nicotinamide adenine dinucleotide; TCA, tricarboxylic acid cycle.

Figure 6A shows a schematic representation of the metabolic changes induced by LDHA inhibitor in Snu398 cells. One of the largest increases seen in cellular metabolites was for the isobar of glucose-1,6-bisphosphate and FBP. Because FBP has been shown to potentiate PKM2 activity [28], we investigated if LDHA inhibitors cause PKM2 activation. Indeed we found that Compound 1 dose dependently increased PK activity in Snu398 but not HepG2 cells (Figure 6B). Compound 1 also dose dependently promoted formation of the active tetrameric form of PKM2 from the inactive monomer (Figure 6C).

### Quinoline 3-sulfonamides inhibit survival of Snu398 cells

**Figure 7:**
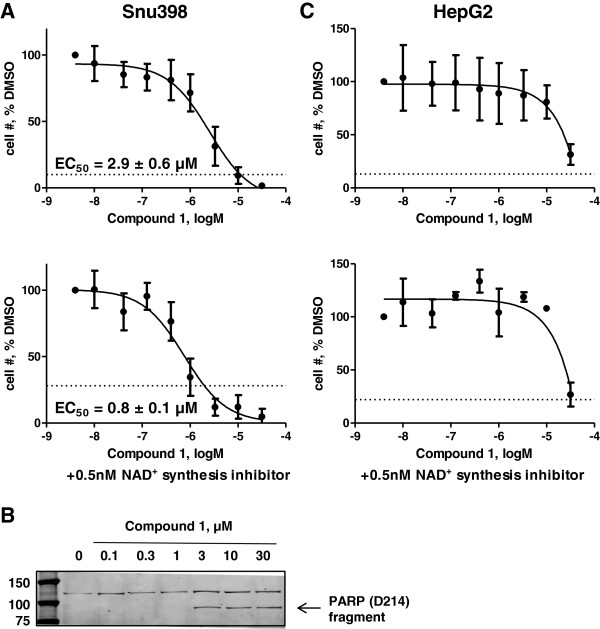
**Compound 1 inhibits cell proliferation and induces apoptosis in Snu398, but not HepG2 human hepatocellular carcinoma cells. (A,C)** Both cell lines were plated in 6-well plates and treated with dimethyl sulfoxide (DMSO) or increasing doses of Compound 1 for 4 to 8 days. At the end of this incubation, numbers of viable cells were assessed by trypan blue exclusion and plotted as a function of Compound 1 concentration. The graphs on the bottom panels were obtained in the presence of 0.5 nM of the nicotinamide adenine dinucleotide (NAD^+^) synthesis inhibitor, FK866. Starting cell densities are indicated by dashed lines. Data are means ± SD of at least four independent experiments except in **(C)**, bottom, where n = 2. **(B)** Snu398 cells were treated with DMSO or increasing doses of Compound 1 for 24 h, whole-cell extracts were isolated and subjected to western immunoblotting with the antibody-recognizing poly (ADP-ribose) polymerase (PARP) (D214) fragment.

We next asked if the reversal of the Warburg metabolic phenotype in Snu398 cells impairs cell survival. We incubated Snu398 cells with increasing doses of Compound 1 for 4 to 8 days and then counted viable cells on the Vi-Cell Analyzer with trypan blue exclusion. Compound 1 dose dependently inhibited proliferation of Snu398 cells (Figure 7A, top panel) and induced apoptosis in Snu398 cells after 24 h of incubation as determined by PARP cleavage observed at doses of 3 to 30 μM (Figure 7B). Since NAD^+^ synthesis is one of the key components of LDHA function, we asked if we can potentiate effects of LDHA inhibition on cell survival by further reducing cellular NAD^+^ supplies. Indeed, addition of the nicotinamide phosphoribosyltransferase (NAMPT) inhibitor of NAD^+^ synthesis, FK866 [29], increased Compound 1 potency and made a 3-μM dose sufficient to induce cell death (Figure 7A, bottom panel). Incubating Snu398 cells in low oxygen conditions (1% O_2_) did not potentiate the Compound 1 effect on cell counts (Additional file 1: Figure S7A). Consistent with its inability to cause long-term metabolic changes in HepG2 cells, Compound 1 did not change HepG2 survival except at the highest dose (Figure 7C, top panel). Switching the cultures to 1% oxygen or adding NAD^+^ synthesis inhibitor did not improve Compound 1 potency in HepG2 cells (Figure 7C bottom panel and Additional file 1: Figure S7B). Direct mitochondrial effects of Compound 1 at 10 μM did not appear to affect survival of HepG2 cells, suggesting that effects on survival in Snu398 cells may indeed be mediated by LDHA inhibition.

### Pharmacokinetic parameters of quinoline-3-sulfonamides

Clearance following IV infusion of Compound 1 at 0.25 mg/kg was shown to be 69 mL/minute/kg in rats, which exceeds the animal liver blood flow [30]. Oral dosing of Compound 1 at 50 mg/kg in rats or 100 mg/kg in mice resulted in blood compound levels at or below the detection limit of 2.5 ng/mL. Due to this low exposure and inability to achieve levels that would be expected to inhibit LDH in animals, no further animal work was conducted with Compound 1.

## Discussion

Potent and selective inhibitors of LDH have been sought for over a decade. However, so far these efforts have resulted in compounds of very modest potency [8,15-18,31], which makes it challenging to correlate the observed cellular effects to LDHA inhibition. Recently sub-micromolar inhibitors have been described but their cellular EC_50_ for lactate inhibition was 200 μM [32]. We report here discovery and development of quinoline 3-sulfonamides that inhibit LDHA enzyme with single-digit nanomolar potency. These compounds exhibit 10- to 80-fold selectivity over the LDHB isoform and do not possess any appreciable activity against a panel of common enzymes, receptors, and ion channels. It is notable that we were able to achieve LDHA selectivity over LDHB, given that the co-factor pocket where our quinoline-3-sulfonamides bind differ only in two conservative changes (Ala98-Val98 and Ile116-Val117 in LDHA and LDHB, respectively). The crystal structures of compounds bound to LDHA demonstrate binding in the NADH pocket only and these compounds are not competitive versus pyruvate (Duffy *et al*. in preparation).

Unfortunately, the optimization of both potency and selectivity towards this challenging target turned out to be incompatible with oral bioavailability and low *in vivo* clearance for this chemical class. As a result, the pharmacokinetic properties of these quinoline 3-sulfonamides are unacceptable for *in vivo* use. Another limitation of these compounds is that at doses of 10 μM and higher they exhibit direct mitochondrial effects that are likely not mediated by LDH inhibition. Nonetheless, these new chemical tools allowed us to investigate several key aspects of LDH biology that have been impossible to address before.

The role of LDHA has been investigated to date mostly using protein level modulation. We found that down-regulation of LDHA protein levels by si- or shRNA in cell lines takes about 5 to 7 days (Additional file 1: Figure S4E), much exceeding the timeline for metabolic processes that happen in the order of minutes. This long gradual down-regulation should allow the cells to adapt their metabolism. Furthermore, down-regulation of the entire protein is not equivalent to only lowering its enzymatic activity. Our small-molecule inhibitors allowed us to avoid these problems and to address consequences of rapid chemical inhibition of LDHA in cancer cells.

Quinoline 3-sulfonamides not only penetrated the cellular membrane but appeared to concentrate inside of cells, possibly due to high protein binding of the compounds or an active transport mechanism for these compounds present in hepatocellular carcinoma cells. Inhibition of LDHA enzymatic activity resulted in profound reduction of lactate production in a variety of cancer cells of different origins. However, low nanomolar inhibitors of the enzyme had EC_50_ no better than 400 nM when tested in cells, which could be explained by micromolar levels of LDHA found in the majority of cancer cell lines tested. These protein concentrations are high, but not unprecedented. Using SILAC-based quantitation in Hek293 cells, Zeiler *et al*. reported several proteins with copy numbers in the millions, including several metabolic enzymes [33]. In fact, LDHA was the 47th protein in abundance in Snu398 cells. Human primary tumors of multiple origins also exhibited LDHA levels comparable to those found in Snu398 cells. In addition to being highly expressed in tumors, LDHA also exhibits rapid kinetics (k_cat_ = 260 s^-1^) [34]. It remains to be seen if such high concentrations of target protein will preclude development of LDHA inhibitors into therapeutics with acceptable safety-to-efficacy ratios.

Overall, sensitivity of cells to LDH inhibition as measured by lactate flux varied greatly between cell lines and did not correlate with levels of either LDHA or LDHB expression (Figure 1C), except in breast cancer (Figure 2D). The reasons for different sensitivity of non-breast cancer lines remain to be investigated and may include differential expression of monocarboxylic transporters, Compound 1 transporters, or various metabolic enzymes.

Two of the most sensitive cell lines - HepG2 and Snu398 hepatocellular carcinoma cells - were selected for further investigation of the consequences of LDHA inhibition. LDHA inhibitors rapidly inhibited lactate production in both cell lines, but only affected glucose consumption in Snu398 cells, suggesting that HepG2 cells can more readily process glucose into metabolites other than lactate. Indeed, we saw, for example, a greater increase in OCR in HepG2 cells upon LDHA inhibition. Snu398, but not HepG2 cells responded to Compound 1 with profound changes in cell metabolism and decreased cell proliferation and survival. Because Compound 1 directly inhibited mitochondrial function at doses ≥10 μM, it is possible that the observed distal metabolic changes, inhibition of cell survival, and induction of apoptosis are not mediated entirely through LDHA inhibition. However, the majority of the metabolic effects observed after 24 h incubation with our LDHA inhibitor are the ones that we could have theoretically predicted due to LDHA inhibition. Evaluation of gene expression profiles of Snu398 and HepG2 cells revealed that Snu398 cells have higher levels of cytosolic glycolysis and pentose phosphate pathway, whereas HepG2 cells preferentially express genes that are involved in fatty acid synthesis and glutaminolysis (Additional file 1: Figure S8A). These opposing metabolic preferences may explain the difference in response of these lines to LDHA inhibition and the ability of HepG2 cells to circumvent it. Another way to address basic metabolic differences between Snu398 and HepG2 cells is to calculate the relative contribution of mitochondrial metabolism and cytosolic anaerobic glycolysis to ATP production. Using the data obtained by Seahorse analysis (Figure 4B), mitochondrial ATP-synthase contribution to OCR can be estimated by subtracting OCR before and after oligomycin addition. Mitochondrial metabolism represents significantly higher contribution to overall ATP production in HepG2 than in Snu398 cells (Additional file 1: Figure S8B). Therefore, because the cells were more dependent on mitochondrial function for ATP production, HepG2 may be expected to more readily compensate and survive if lactate production is inhibited.

In addition to changing the metabolic profile of Snu398 cells, LDHA inhibition resulted in increased PKM2 tetramerization and activity. This effect may be mediated by an increase in FBP, as FBP has been shown to increase PKM2 activity [28] and as LDHA inhibition in HepG2 cells does not lead to increases in FBP or PK activity (Additional file 4: Table S3 and Figure 6B). PKM2 is highly expressed in cancer cells, but not in normal adult cells, and is biochemically less active than PKM1 [35]. Replacing PKM2 with the more active PKM1 isozyme in human cancer cells fails to support the Warburg effect and inhibits tumor formation [36,37], and PKM2 activators suppress tumorigenesis [38]. Thus, increasing PKM2 activation could provide yet another mechanism for LDHA inhibitors to reduce tumor growth. On the other hand, upon activation by the EGF receptor (EGFR), PKM2 has been shown to translocate to the nucleus and act as a transcription coactivator to increase c-myc and LDHA expression [39], suggesting a possible escape mechanism for LDHA inhibition that remains to be further investigated.

## Conclusions

We developed a series of quinoline 3-sulfonamides that potently and selectively inhibited LDHA and led to a rapid reduction of glucose uptake and lactate production. LDHA inhibition resulted in profound changes in overall metabolism and survival in hepatocellular carcinoma cells.

## Abbreviations

ANOVA: Analysis of variance; ATCC: American type culture collection; BCA: Bicinchoninic acid; DMSO: Dimethyl sulfoxide; EC50: Half maximal effective concentration; ECACC: European collection of cell cultures; ECAR: Extracellular acidification rate; EGF: Epidermal growth factor; EGFR: Epidermal growth factor receptor; DMEM: Dulbecco’s modified Eagle's medium; FBP: Fructose-1,6-bisphosphate; FBS: Fetal bovine serum; GSK: GlaxoSmithKline; IV: Intravenous; LC: Liquid chromatography; LDH: Lactate dehydrogenase; MS: Mass spectrometry; NAD+: Nicotinamide adenine dinucleotide; NCI: National cancer institute; NMR: Nuclear magnetic resonance; OCR: Oxygen consumption rate; OCROLG: Oligomycin-dependent oxygen consumption rate; PARP: Poly (ADP-ribose) polymerase; PBS: Phosphate-buffered saline; PK: Pyruvate kinase; PKM1: Pyruvate kinase M1; PVDF: Polyvinylidene fluoride; RIPA: Radioimmunoprecipitation assay; RPMI: Roswell park memorial institute medium; SILAC: Stable isotope labeling by amino acids in cell culture; TCA: Tricarboxylic acid cycle.

## Competing interests

Julia Billiard, Roland S Annan, Jacques Briand, Deping Chai, Mariela Colón, Christopher S Dodson, Kevin J Duffy, Seth Gilbert, Joel Greshock, Junping Jing, Hong Lu, Lisa A Orband-Miller, Chad Quinn, Jessica L Schneck, Gilbert F Scott, Anthony N Shaw, James P Villa, Gregory Waitt, and Richard F Wooster are employees of GlaxoSmithKline; Gordon B Mills and Jennifer B Dennison receive sponsored research support from GlaxoSmithKline.

## Authors’ contributions

JB, JD, JBr, and RA performed experiments and wrote the manuscript; DC, MC, CD, SG, JG, JJ, HL, JMF, LOM, CQ, JS, GS, AS, and GW performed acquisition and analysis of data; GM, RW, and KD conceptually guided the project and revised the manuscript for important intellectual content. All authors read and approved the final manuscript.

## Supplementary Material

Additional file 1: Figures S1-S7 Figure legends and Additional Methods are listed in Additional file 6. Click here for file

Additional file 2: Table S1 Quinoline 3-sulfonamides do not exhibit appreciable activity against a panel of approximately 32 common enzymes, receptors, and ion channels. ANT = antagonist (pIC_50_), AG = agonist (pEC_50_). pIC_50_ and pEC_50_ are the negative logarithms_10_ of IC_50_ and EC_50_, respectively. Click here for file

Additional file 3: Table S2 Tissue origin and lactate dehydrogenase A (LDHA) and LDHB transcript expression levels for cancer cell lines used in Figure 1. Transcript expression levels in MAS5 units were obtained from gene expression analysis data for 428 cancer cell lines that we previously submitted to public database (https://cabig-stage.nci.nih.gov/community/caArray_GSKdata/). MAS5 units are obtained by processing the data using Affymetrix MAS5 algorithm, with target value set at 100. Click here for file

Additional file 4: Table S3 Comprehensive metabolic analysis of Snu398 and HepG2 cellular extracts upon addition of lactate dehydrogenase A (LDHA) inhibitor. Cells were treated with 10 μM of Compound 2 or dimethyl sulfoxide (DMSO) control for 24 h and whole-cell extracts were subjected to mass spectrometry analysis that detected 577 metabolites. Ion counts of five replicates per condition obtained before and after compound addition were averaged and fold change upon compound addition was calculated. The Table lists fold changes and *P*-values for all detected metabolites. Statistically significant (*P* <0.05) increases are highlighted in red and decreases, in green. Click here for file

Additional file 5: Table S4 Comprehensive metabolic analysis of Snu398 and HepG2 conditioned media upon addition of lactate dehydrogenase A (LDHA) inhibitor. Cells were treated with 10 μM of Compound 2 or dimethyl sulfoxide (DMSO) control for 24 h and conditioned media were subjected to mass spectrometry analysis that detected 227 metabolites. Ion counts of five replicates per condition obtained before and after compound addition were averaged and fold change upon compound addition was calculated. The Table lists fold changes and *P*-values for all detected metabolites. Statistically significant (*P* <0.05) increases are highlighted in red and decreases, in green. Click here for file

Additional file 6 Additional Methods and Figure Legends. Click here for file
