# Supplementary material for: Quinoline 3-sulfonamides inhibit lactate dehydrogenase A and reverse aerobic glycolysis in cancer cells
Source: Cancer Metab. 2013 Sep 6;1:19. doi: 10.1186/2049-3002-1-19 (PMC4178217; doi:10.1186/2049-3002-1-19)
Supplement: Additional file 1: Figures S1-S7 — Figure legends and Additional Methods are listed in Additional file 6. [file 2049-3002-1-19-S1.pptx]

## Slide 1
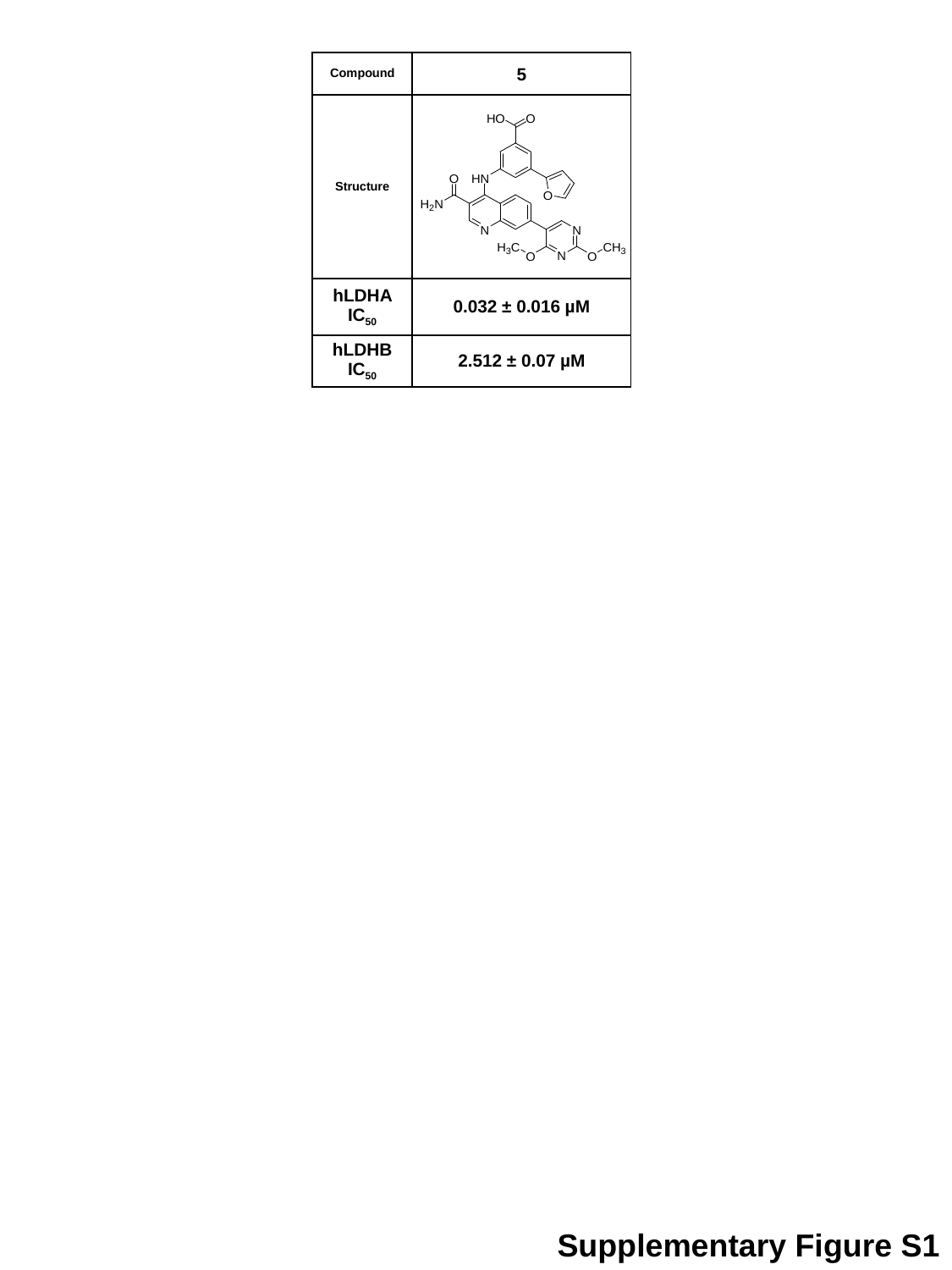

| Compound | 5 |
| --- | --- |
| Structure | |
| hLDHA IC50 | 0.032 ± 0.016 µM |
| hLDHB IC50 | 2.512 ± 0.07 µM |
Supplementary Figure S1

## Slide 2
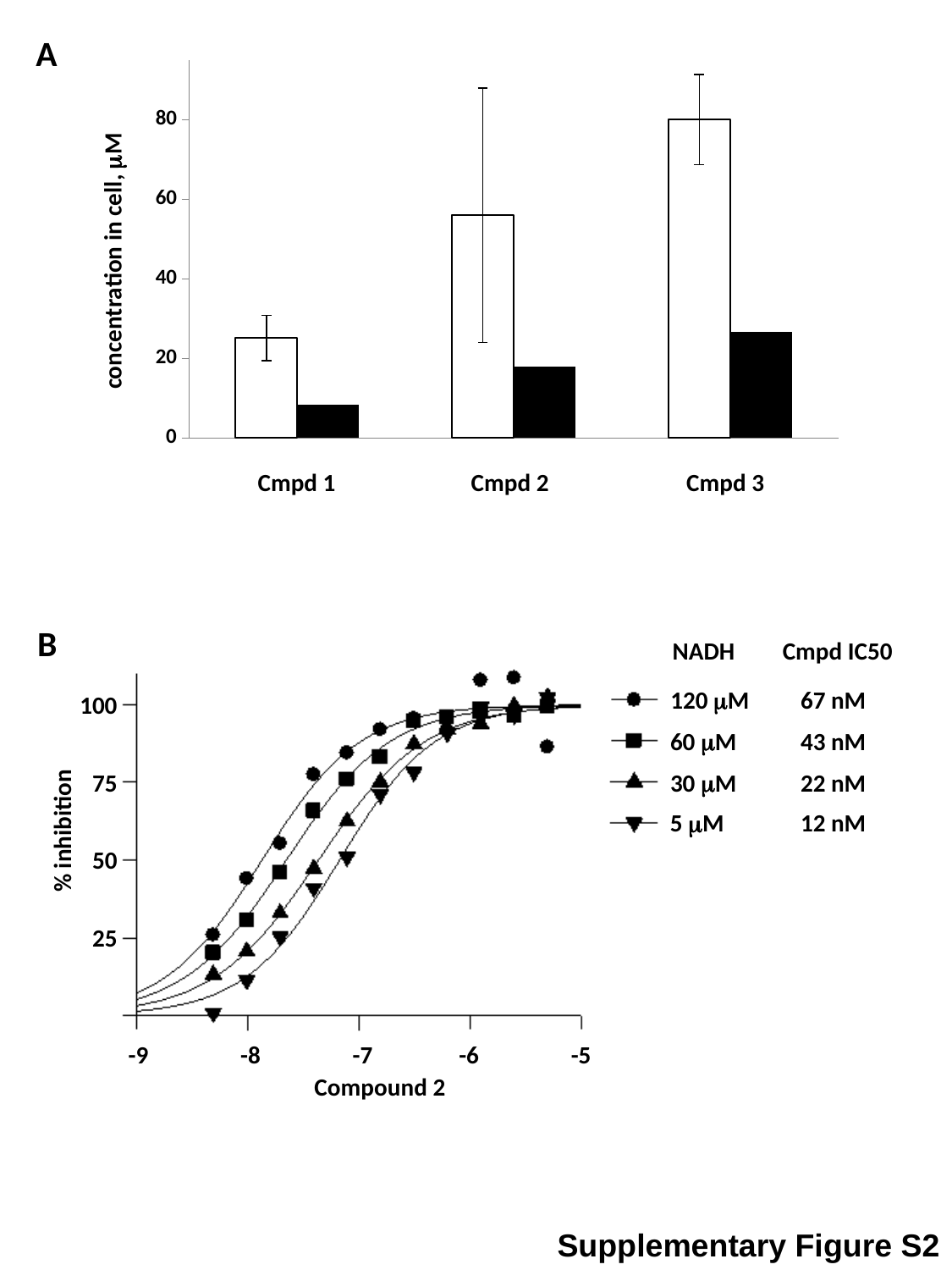

A
### Chart
| Category | | |
|---|---|---|concentration in cell, mM
Cmpd 1
Cmpd 2
Cmpd 3
B
NADH
Cmpd IC50
120 mM
67 nM
100
60 mM
43 nM
30 mM
22 nM
75
5 mM
12 nM
% inhibition
50
25
-9
-8
-7
-6
-5
Compound 2
Supplementary Figure S2

## Slide 3
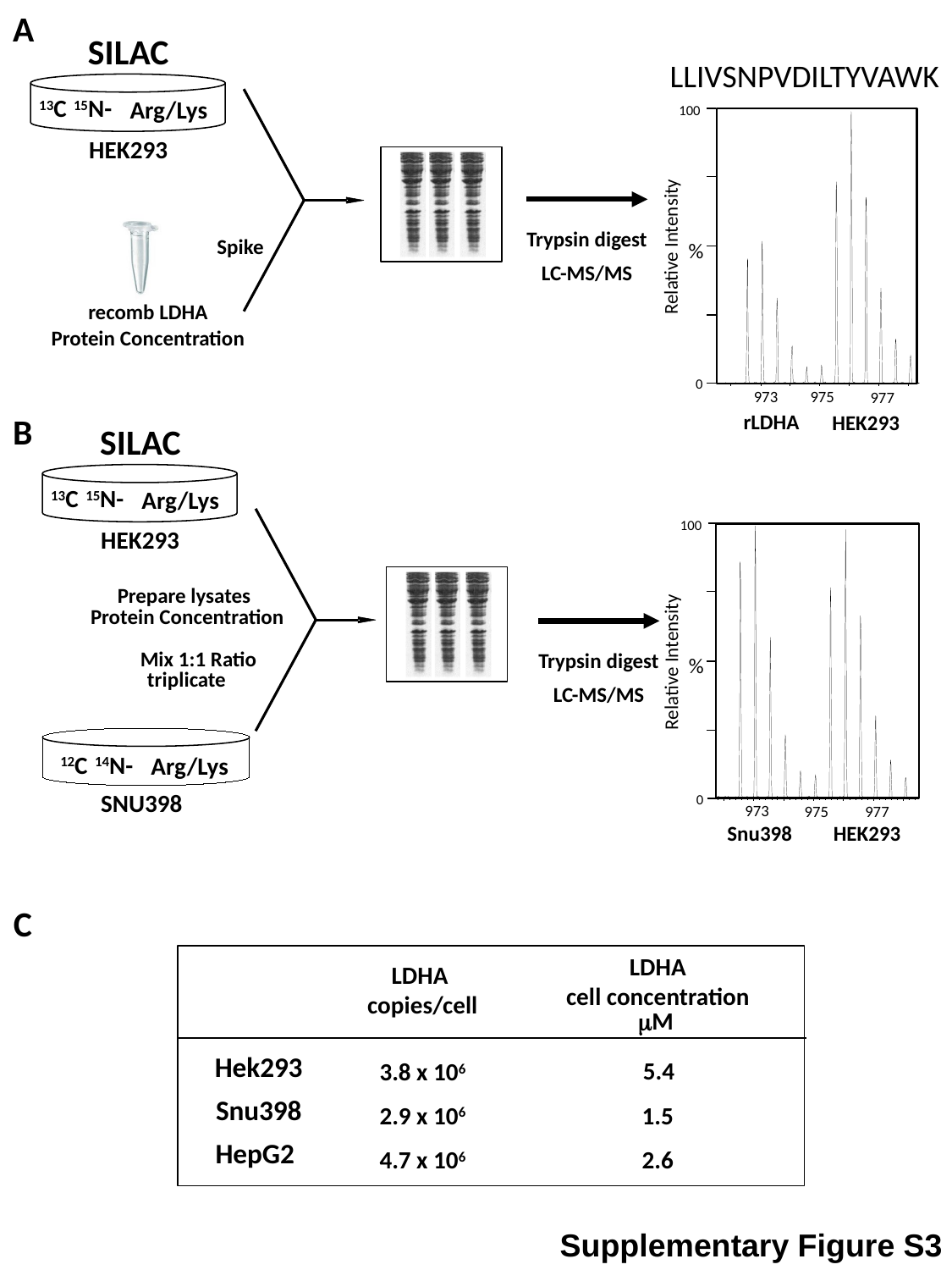

A
SILAC
LLIVSNPVDILTYVAWK
100
%
Relative Intensity
0
973
975
977
rLDHA
HEK293
13C
 15N-
Arg/Lys
HEK293
Trypsin digest
LC-MS/MS
Spike
recomb LDHA
Protein Concentration
B
SILAC
13C
 15N-
Arg/Lys
100
%
Relative Intensity
0
973
975
977
Snu398
HEK293
HEK293
Prepare lysates
Protein Concentration
Trypsin digest
LC-MS/MS
Mix 1:1 Ratio
triplicate
12C
 14N-
Arg/Lys
SNU398
C
LDHA
cell concentration
LDHA
copies/cell
mM
Hek293
5.4
3.8 x 10^6^
Snu398
2.9 x 10^6^
1.5
HepG2
4.7 x 10^6^
2.6
Supplementary Figure S3

## Slide 4
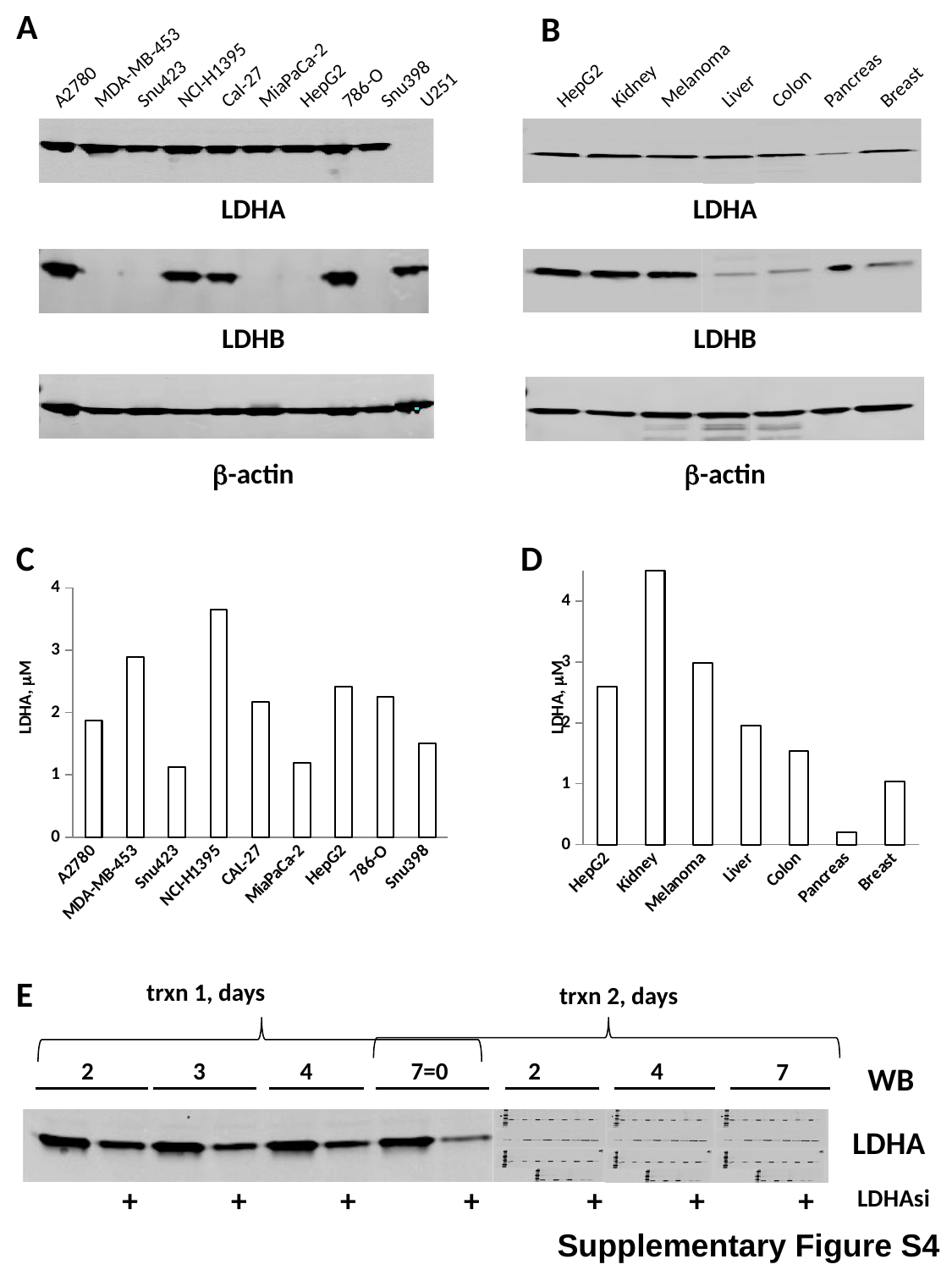

B
A
A2780
MDA-MB-453
Snu423
NCI-H1395
Cal-27
MiaPaCa-2
HepG2
786-O
Snu398
U251
HepG2
Kidney
Melanoma
Liver
Colon
Pancreas
Breast
LDHA
LDHA
LDHB
LDHB
b-actin
b-actin
C
D
### Chart
| Category | |
|---|---|
| HepG2 | 2.6 |
| Kidney | 4.498 |
| Melanoma | 2.9899999999999998 |
| Liver | 1.9500000000000033 |
| Colon | 1.534 |
| Pancreas | 0.2080000000000002 |
| Breast | 1.04 |
### Chart
| Category | |
|---|---|
| A2780 | 1.8741536713280431 |
| MDA-MB-453 | 2.88893850137166 |
| Snu423 | 1.1206909143277881 |
| NCI-H1395 | 3.6488582759061288 |
| CAL-27 | 2.177604545939784 |
| MiaPaCa-2 | 1.1954042731075358 |
| HepG2 | 2.4119427012684977 |
| 786-O | 2.250616820499213 |
| Snu398 | 1.5 |LDHA, mM
LDHA, mM
E
trxn 1, days
trxn 2, days
2
3
4
7=0
2
4
7
WB
LDHA
+ + + + + + +
LDHAsi
Supplementary Figure S4

## Slide 5
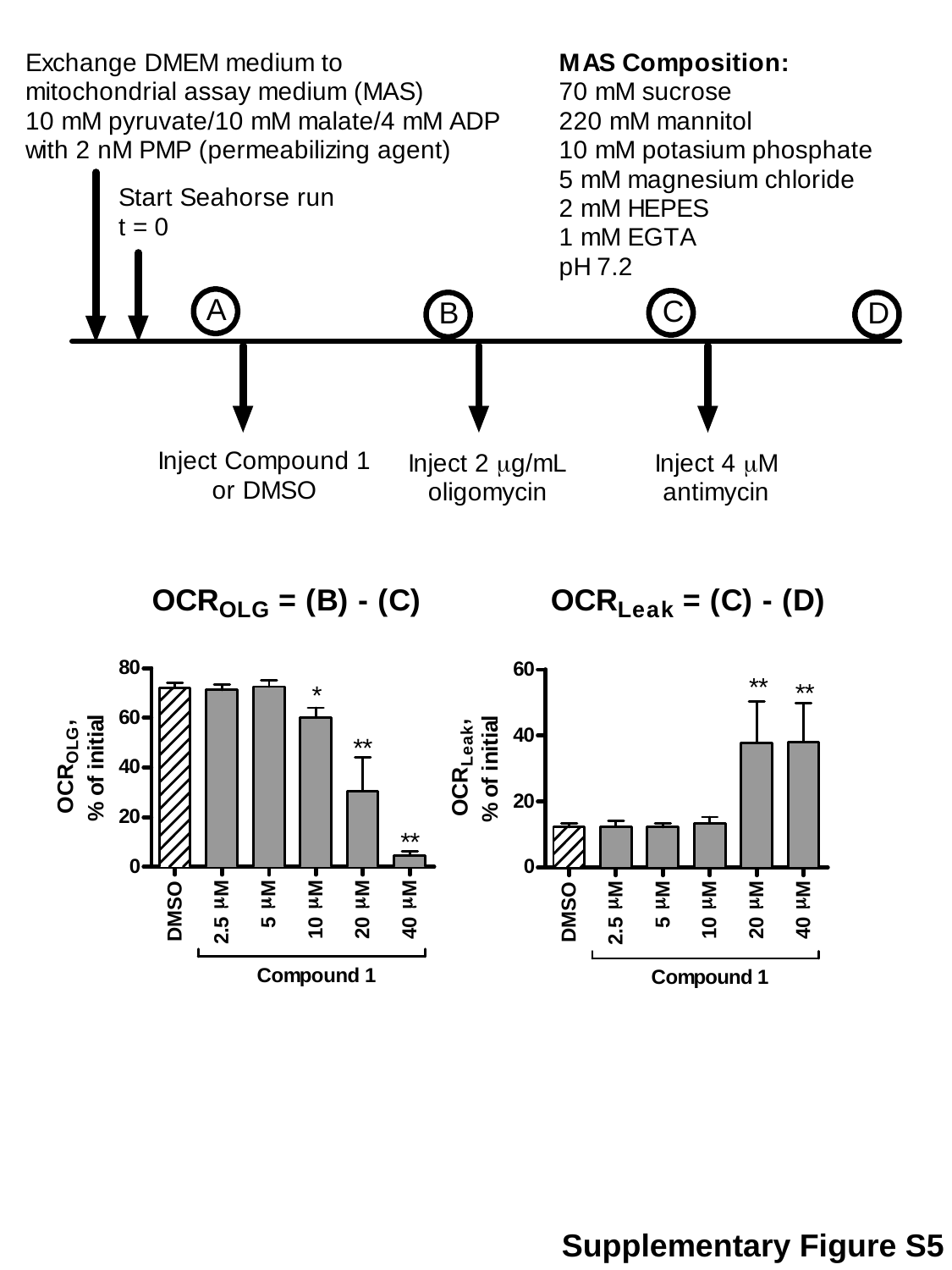

Supplementary Figure S5

## Slide 6
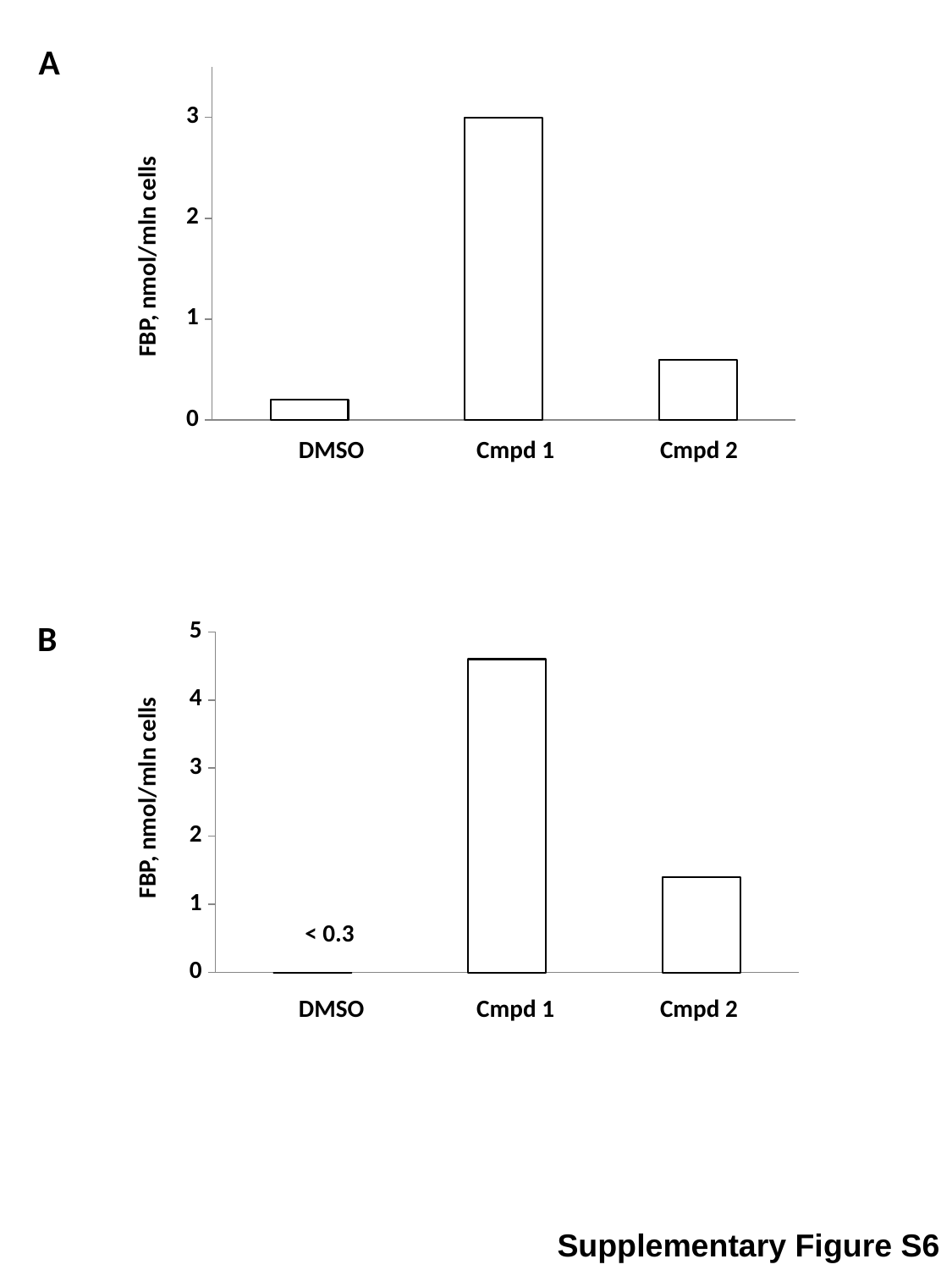

A
### Chart
| Category | |
|---|---|FBP, nmol/mln cells
DMSO
Cmpd 1
Cmpd 2
B
### Chart
| Category | |
|---|---|FBP, nmol/mln cells
< 0.3
DMSO
Cmpd 1
Cmpd 2
Supplementary Figure S6

## Slide 7
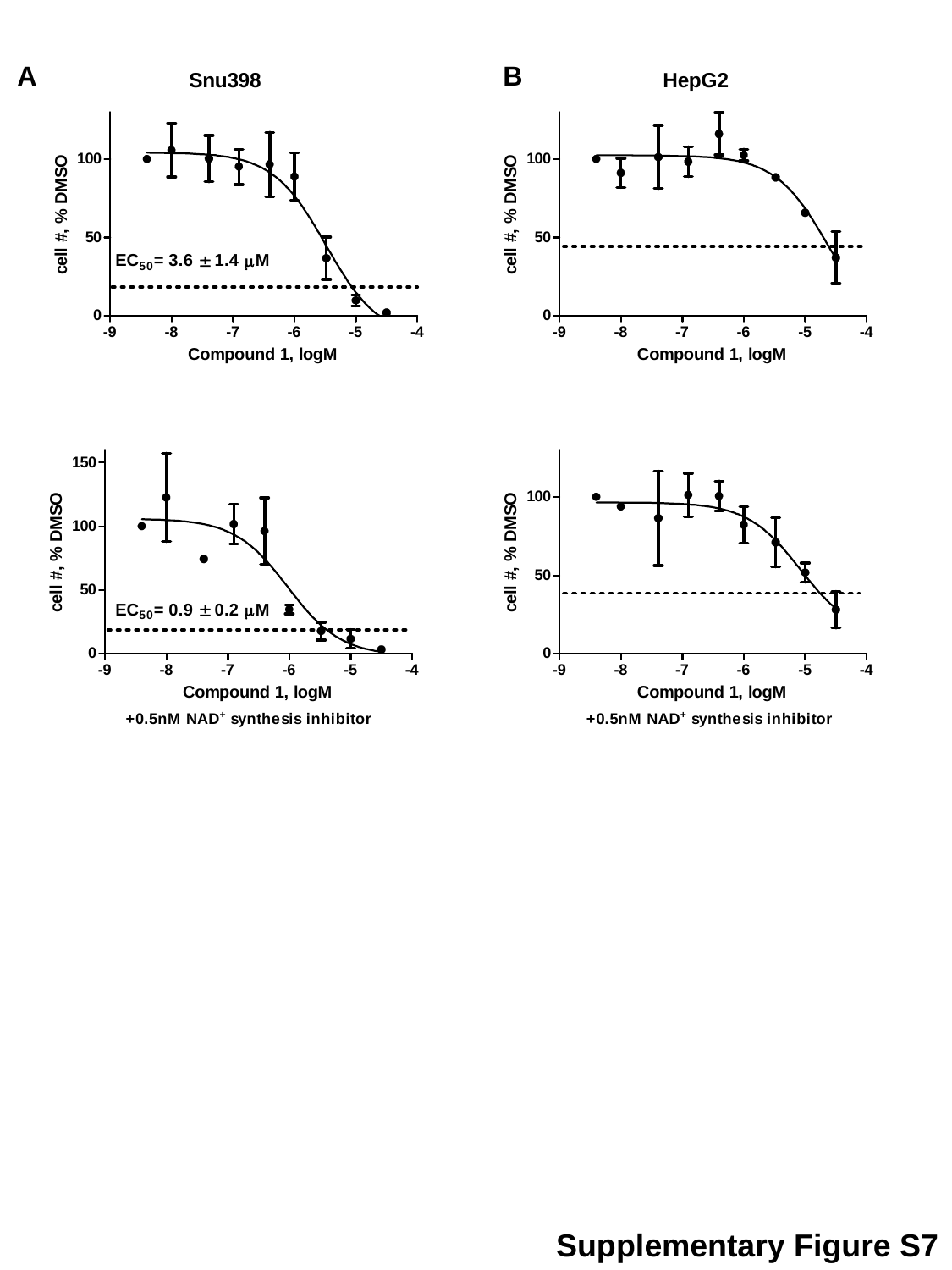

Supplementary Figure S7

## Slide 8
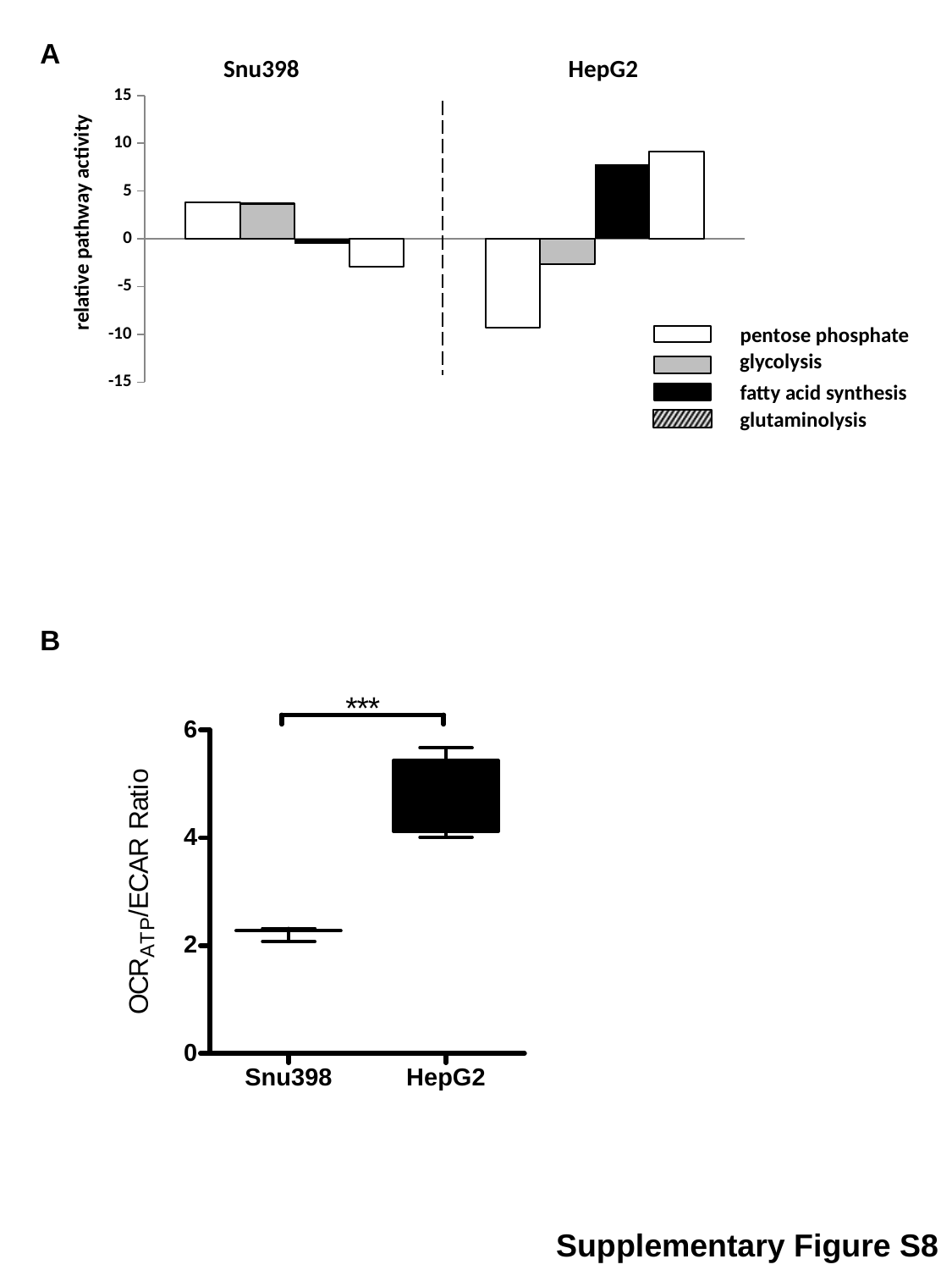

A
Snu398
HepG2
### Chart
| Category | | | | |
|---|---|---|---|---|relative pathway activity
pentose phosphate
glycolysis
fatty acid synthesis
glutaminolysis
B
Supplementary Figure S8
