# Supplementary material for: Quinoline 3-sulfonamides inhibit lactate dehydrogenase A and reverse aerobic glycolysis in cancer cells
Source: Cancer Metab. 2013 Sep 6;1:19. doi: 10.1186/2049-3002-1-19 (PMC4178217; doi:10.1186/2049-3002-1-19)
Supplement: Additional file 6 — Additional Methods and Figure Legends. [file 2049-3002-1-19-S6.docx]

**Additional Methods**

***Compound cellular permeability assays*** For cytosolic permeability assay, twelve million Snu398 cells were allowed to attach overnight and treated with DMSO control or 1 μM Compound 2 for 1.5 h in 1.7 mM glucose medium. The medium was removed, cells were washed in phosphate-buffered saline (PBS), and cytosolic extracts were prepared by solubilization in Qproteome lysis buffer (Qiagen, Valencia, CA). For whole-cell permeability assay, 75,000 Snu398 cells/well were plated in 96-well plates and treated with DMSO control or 1 μM compounds for 1.5h in low glucose medium. The medium was removed, cells were washed in PBS and solubilized in 50:50 mixture of methanol and acetonitrile. Compound concentration in the cytosolic or whole-cell extract was determined by LC/MS/MS analysis. An Acquity UPLC system (Waters Inc., Milford, MA) was coupled to an API 4000 or API 5000 triple-quad mass spectrometer (Applied Biosystems).  A gradient from 5% to 95% organic over 1 minute or less was used to separate the compound of interest and the appropriate negative or positive MS/MS transition was used to detect the analyte response. Compound concentration in cells was determined by counting the number of cells in sample wells and assuming 3 pL/cell volume.

***LDHA enzymatic assay with increasing levels of NADH*** The following reaction was assembled in clear-bottom 96 well plate: 50 mM phosphate buffer pH 7.0, 50 mM NaCl, 0.25 mM CHAPS, 200 μM NaPyruvate, 250 pM recombinant human LDHA, and the indicated amounts of NADH and Compound 2. Absorbance change due to NADH conversion to NAD^+^ was read on SpectraMax M2 (Molecular Devices, Sunnyvale, CA) at 340 nm every 40 sec for 6 min.

***Absolute SILAC analysis*** The method was modified from ^[1]^ and is schematically represented in Supplementary Fig. S2. Hek293 cells were grown in L-arginine-U-^13^C_6_-^15^N_4_ and L-lysine-U-^13^C_6_-^15^N_2_ for six doubling in advanced D-MEM/F12-Flex SILAC medium (Invitrogen). Cells (4.2x10e7) were lysed in RIPA buffer (Sigma) containing HALT protease inhibitor cocktail (Thermo Scientific), mixed with increasing doses of recombinant human full-length LDHA protein and separated by SDS-PAGE gel electrophoresis. A section of each lane corresponding to elution position of LDHA was excised, reduced, alkylated, and digested with trypsin. Tryptic digests were analyzed by data dependent LC-MS/MS on an Orbitrap XL mass spectrometer. Protein identification and LDHA protein ratios were determined using the MaxQuant quantitative proteomics software. Snu398 or HepG2 cells were lysed in RIPA buffer with HALT protease inhibitor cocktail. SILAC labeled Hek293 lysate was mixed 1:1 by weight with Snu398 or HepG2 lysate and separated by SDS-PAGE gel electrophoresis. A gel section corresponding to the elution position of LDHA was excised from each lane and processed as described above. The obtained protein ratios (Hek293/Snu398=1.3 and Hek293/HepG2=0.8) were used to determine the LDHA copy number in the respective cancer cell line. Cell volumes were calculated for all lines using cell diameter determined by Vi-Cell XR Cell Viability Analyzer and that value was used to convert copy number to concentration. The volume was 1.15 pL for Hek293 cells and 3 pL for Snu398 and HepG2 cells.

***LDH Western immunoblotting*** The human biological samples were sourced ethically and their research use was in accord with the terms of the informed consents. Whole-cell proteins were isolated from the indicated cell lines or frozen human tumor samples of indicated origin using RIPA buffer with protease inhibitor cocktail and subjected to Western immunoblotting with LDHA, LDHB, or β-actin-specific antibodies or HepG2 whole-cell protein. Twenty μg of whole-cell extracts were run alongside 20 μg of Snu398 extract and 14 μg of tumor lysates were used alongside 14 μg of HepG2 extract.

***Transfections with siRNA*** HPAC human pancreatic adenocarcinoma cells were reverse transfected in R10 medium using 20nM of On-Target Plus LDHA siRNA #4 or On-Target Plus control pool siRNA (Thermo Fisher Scientific) and 2.5 μL/mL Lipofectamine RNAiMAX reagent (Life Technologies). Cells were allowed to proliferate for the indicated times prior to protein isolation. After 7 days of incubation, cells were lifted by trypsinization, re-transfected with the same siRNA by the same method, re-plated into 6-well plates, and allowed to proliferate for the indicated times prior to protein isolation and Western immunoblotting.

***Fructose-1,6-bisphosphate (FBP) analysis*** Two million Snu398 cells were plated in T150 flasks, allowed to attach for 18 h, and treated with DMSO control or 10 μM of Compound 1 or 2. Treatment was carried out for 24 h in R10 or for 6 h in low glucose medium. At the end of this treatment, cells were washed, lifted by trypsinization, counted, and cell pellets were submitted for analysis of FBP to Apredica, Inc.

**Additional Figure Legends**

**Additional Figure S1.** Lactate dehydrogenase A (LDHA) inhibitor with 80-fold selectivity for LDHA over LDHB enzyme.

**Additional Figure S2.** (A) Quinoline 3-sulfonamides have higher concentrations in Snu398 cellular extracts than in the medium. Snu398 cells were treated with 1 μM Compounds 1-3 for 1.5 h, and compound concentrations inside whole-cell extracts were assessed by liquid chromatography (LC)/mass spectrometry (MS)/MS analysis (open bars). This method does not distinguish between compound bound to cellular membranes and compound in the cytosol. Concentration of Compound 2 in the cytosol upon cellular fractionation was found to be 18 μM (see main text) which corresponds to 1/3 of the concentration in whole-cell extract. This ratio was used to estimate cytosolic levels for the other Compounds (closed bars). (B) Activity of Compound 2 in LDHA enzymatic assay performed at different levels of NADH. NADH concentrations and the corresponding Compound 2 IC_50_s are shown on the right.

**Additional Figure S3.** Concentration of LDHA in Snu398 and HepG2 cells. (A) Schematic representation of determining LDHA concentration in Hek293 cells using absolute SILAC method. (B) Schematic representation of determining LDHA concentration in Snu398 cells. (C) The resultant LDHA copy number and concentration in Hek293, Snu398 and HepG2 cells. The concentration was calculated from the copy number using cell volume of 1.15 pL for Hek293 cells and 3 pL for Snu398 and HepG2 cells. The cell volumes were calculated using cell diameter determined by Vi-Cell XR Cell Viability Analyzer.

**Additional Figure S4.** Concentration of LDHA in cancer cells and tissues. (A) Western immunoblotting confirms relative LDH expression levels obtained from gene expression analysis. (B) Western immunoblotting of LDH expression levels in cancer tissues. (C, D) Quantification of western immunoblotting demonstrates that majority of cancer cell lines and tissues express micromolar levels of LDHA. Densitometry was performed on all LDHA and β-actin bands, LDHA values were normalized to β-actin and compared to the LDHA/β-actin ratio for Snu398 (C) or HepG2 (D) cells. (E) Down-regulation of LDHA expression by siRNA in HPAC cells takes 5-7 days. Cells were transfected (trxn 1) with LDHA siRNA (+) or non-targeting control siRNA, and incubated for the indicated number of days. Seven days after transfection 1, cells were re-transfected (trxn 2), replated and allowed to proliferate for the indicated times prior to protein isolation and western immunoblotting.

**Additional Figure S5.** Direct mitochondrial effects of Compound 1 using permeabilized cells. Oxygen consumption rate (OCR) readings A through D were obtained before and after drug injections. The absolute OCR reduction after oligomycin addition was defined at the ATP-dependent OCR (OCR_OLG_). The OCR from mitochondrial proton leak (OCR_Leak_) was defined as the residual OCR after oligomycin addition less the non-mitochondrial OCR determined by antimycin addition. **P* <0.05, ***P* <0.01

**Additional Figure S6.** Compounds 1 and 2 increase intracellular levels of fructose-1,6-bisphosphate (FBP) in Snu398 cells. Snu398 cells were treated with dimethyl sulfoxide (DMSO) control or 10 μM of Compound 1 or 2 either in R10 medium for 24 h (A) or in low glucose medium for 6 h (B). Cells were washed, lifted by trypsinization, counted, and cell pellets were submitted for analysis of FBP to Apredica, Inc. In (B), the level of FBP activity in untreated cells was below detection limit of approximately 0.3 nmol/million cells. Since each experiment was performed once, both are included to demonstrate that Compound 1 increases FBP activity similar to or better than Compound 2.

**Additional Figure S7.** Compound 1 inhibits cell proliferation and induces apoptosis in Snu398 (A), but not HepG2 (B), human hepatocellular carcinoma cells in 1% oxygen conditions. Both cell lines were plated in 6-well plates and treated with DMSO or increasing doses of Compound 1 for 4 to 8 days. At the end of this incubation, numbers of viable cells were assessed by trypan blue exclusion and plotted as a function of Compound 1 concentration. Starting cell densities are indicated by dashed lines. The graphs on the bottom panels were obtained in presence of 0.5 nM of the NAD^+^ synthesis inhibitor, FK866. Data are means ± SD of at least two independent experiments.

**Additional Figure S8.** (A) Different contributions of various metabolic pathways in Snu398 and HepG2 hepatocellular carcinoma cells. Gene expression analysis of previously obtained data (<https://cabig-stage.nci.nih.gov/community/caArray_GSKdata/>) was used to identify contributions of different metabolic signatures. (B) Contributions of mitochondrial metabolism versus cytosolic glycolysis to ATP production in Snu398 and HepG2 cells. The difference in oxygen consumption rate (OCR) before and after oligomycin addition was calculated to estimate mitochondrial contribution to overall ATP production. The ratio of oligomycin-sensitive OCR (OCR_ATP_) to extracellular acidification rate (ECAR) is plotted for Snu398 and HepG2 cells. ****P* < 0.001

**Reference**

1. Ong, S. E., Blagoev, B., Kratchmarova, I., Kristensen, D. B., Steen, H., Pandey, A., and Mann, M.: **Stable isotope labeling by amino acids in cell culture, SILAC, as a simple and accurate approach to expression proteomics.** *Mol Cell Proteomics* 2002, **1**:376-386.
